# Heterogeneity in the progression of retinal pathologies in mice harboring patient mimicking *Impg2* mutations

**DOI:** 10.1093/hmg/ddad199

**Published:** 2023-11-17

**Authors:** Brittany N Williams, Adam Draper, Patrick F Lang, Tylor R Lewis, Audrey L Smith, Steven J Mayerl, Marie Rougié, Jeremy M Simon, Vadim Y Arshavsky, Scott H Greenwald, David M Gamm, Isabel Pinilla, Benjamin D Philpot

**Affiliations:** Neuroscience Center, University of North Carolina, Chapel Hill, NC 27599, United States; Department of Cell Biology and Physiology, University of North Carolina, Chapel Hill, NC 27599, United States; Carolina Institute for Developmental Disabilities, University of North Carolina, Chapel Hill, NC 27599, United States; Neuroscience Center, University of North Carolina, Chapel Hill, NC 27599, United States; Department of Cell Biology and Physiology, University of North Carolina, Chapel Hill, NC 27599, United States; Neuroscience Center, University of North Carolina, Chapel Hill, NC 27599, United States; Department of Cell Biology and Physiology, University of North Carolina, Chapel Hill, NC 27599, United States; Department of Ophthalmology, Duke University, Durham, NC 27705, United States; Neuroscience Center, University of North Carolina, Chapel Hill, NC 27599, United States; Department of Cell Biology and Physiology, University of North Carolina, Chapel Hill, NC 27599, United States; Department of Ophthalmology and Visual Sciences, McPherson Eye Research Institute, University of Wisconsin-Madison, Madison, WI 53705, United States; Neuroscience Center, University of North Carolina, Chapel Hill, NC 27599, United States; Department of Cell Biology and Physiology, University of North Carolina, Chapel Hill, NC 27599, United States; Neuroscience Center, University of North Carolina, Chapel Hill, NC 27599, United States; Carolina Institute for Developmental Disabilities, University of North Carolina, Chapel Hill, NC 27599, United States; Department of Ophthalmology, Duke University, Durham, NC 27705, United States; Kagu Consulting, Cary, NC 27519, United States; Department of Ophthalmology and Visual Sciences, McPherson Eye Research Institute, University of Wisconsin-Madison, Madison, WI 53705, United States; Department of Ophthalmology, Lozano Blesa University Hospital, Zaragoza 50009, Spain; Aragón Health Research Institute (IIS Aragón), Zaragoza 50009, Spain; Department of Surgery, University of Zaragoza, Zaragoza 50009, Spain; Neuroscience Center, University of North Carolina, Chapel Hill, NC 27599, United States; Department of Cell Biology and Physiology, University of North Carolina, Chapel Hill, NC 27599, United States; Carolina Institute for Developmental Disabilities, University of North Carolina, Chapel Hill, NC 27599, United States

**Keywords:** retinitis pigmentosa, IMPG2, cone-rod dystrophy, inherited retinal disorder, interphotoreceptor matrix

## Abstract

Biallelic mutations in interphotoreceptor matrix proteoglycan 2 (*IMPG2*) in humans cause retinitis pigmentosa (RP) with early macular involvement, albeit the disease progression varies widely due to genetic heterogeneity and *IMPG2* mutation type. There are currently no treatments for IMPG2-RP. To aid preclinical studies toward eventual treatments, there is a need to better understand the progression of disease pathology in appropriate animal models. Toward this goal, we developed mouse models with patient mimicking homozygous frameshift (T807Ter) or missense (Y250C) *Impg2* mutations, as well as mice with a homozygous frameshift mutation (Q244Ter) designed to completely prevent IMPG2 protein expression, and characterized the trajectory of their retinal pathologies across postnatal development until late adulthood. We found that the *Impg2*^*T807Ter/T807Ter*^ and *Impg2*^*Q244Ter/Q244Ter*^ mice exhibited early onset gliosis, impaired photoreceptor outer segment maintenance, appearance of subretinal deposits near the optic disc, disruption of the outer retina, and neurosensorial detachment, whereas the *Impg2*^*Y250C/Y250C*^ mice exhibited minimal retinal pathology. These results demonstrate the importance of mutation type in disease progression in IMPG2-RP and provide a toolkit and preclinical data for advancing therapeutic approaches.

## Introduction

The interphotoreceptor matrix (IPM) is a network of proteins in the retina that surrounds the inner and outer segments of rod and cone photoreceptors and extends to the retinal pigment epithelium (RPE) [1]. The IPM serves many functions such as providing structural support to photoreceptors, transport of nutrients to photoreceptors, intercellular communication, retinoid transport between the photoreceptors and RPE, and adhesion of the RPE to the neural retina [1–6]. To accomplish these varied functions, the RPE and photoreceptors synthesize IPM components that integrate into a complex network of extracellular and membrane-bound proteins [1, 7]. These proteins include enzymes, adhesive glycoproteins, growth factors, glycosaminoglycans, hyaluronan-binding proteins, and proteoglycans [1, 8–15]. Two of the most abundant IPM proteins are interphotoreceptor matrix proteoglycan 1 (IMPG1; also known as SPACR and IPM150) and interphotoreceptor matrix proteoglycan 2 (IMPG2; also known as SPACRCAN or IPM200) [16, 17]. IMPG1 and IMPG2 are heavily glycosylated and provide a primary source of chondroitin sulfate in the IPM [15, 18, 19], with an additional 50%–60% mass added to IMPG2 through the posttranslational addition of various carbohydrates [16, 18]. These modifications are thought to support the interconnectivity and structural integrity of the IPM. Given the abundance and central role of *IMPG1* and *IMPG2* in the IPM, it is not surprising that mutations in these genes have been linked to retinal pathology and vision loss [20, 21].

In humans, biallelic *IMPG2* mutations cause retinitis pigmentosa (RP), whereas monoallelic *IMPG2* mutations are linked to adult-onset vitelliform macular dystrophy (AVMD) [22–30]. Stargardt-like macular dystrophy has also been described in individuals with homozygous *IMPG2* mutations [28]. Symptoms of IMPG2-RP include night blindness, retinal thinning, fundus abnormalities, and loss of visual acuity associated with macular atrophy [31]. The age of onset for visual deficits linked to autosomal recessive *IMPG2* mutations varies (~4–20 years), likely due to differences in genetic background and *IMPG2* mutation type, with a mean of around 10.5 years [31]. Unlike the profound visual loss associated with biallelic *IMPG2* mutations, heterozygous *IMPG2* mutations cause much milder visual impairments that generally manifest at a mean onset age of 43 years. Although dominant *IMPG2* mutations are associated with the development of vitelliform macular lesions, patients typically have normal or borderline full-field electroretinogram (ERG) and electrooculogram (EOG) results [21, 32, 33]. There are currently no treatments for any IMPG2-mediated inherited retinal disorders (IRDs).

The postnatal manifestation of retinal pathology in IMPG2-RP suggests it will be amenable to gene editing or gene addition therapeutic interventions — strategies that are facilitated by having relevant patient cell lines and animal models. Toward this goal, patient-derived and gene-edited human induced pluripotent stem cell (hiPSC) and human embryonic stem cell (hESC) lines were recently generated to model compound heterozygous *IMPG2* mutations (*IMPG2^Y254C/A805(fs)ter^*) [34]. Human retinal organoids harboring these patient *IMPG2* mutations were unable to maintain photoreceptor outer segments in a manner that modeled advanced RP [34].

To complement the existing hiPSC and hESC IMPG2 models and to aid preclinical testing of therapeutics, here we generated mice with patient mimicking *Impg2* mutations: mouse T807Ter mutation to mimic the human A805Ter mutation and mouse Y250C mutation to mimic the human Y254C mutation. We found that *Impg2^T807Ter/T807Ter^* mice exhibited rapid onset of gliosis, subretinal deposits, and progressive impairment of photoreceptor maintenance, whereas the *Impg2^Y250C/Y250C^* mice exhibited no easily discernable retinal pathology into late adulthood. Our data highlight the unique disease progression of patient mutations.

## Results

**Figure 1:**
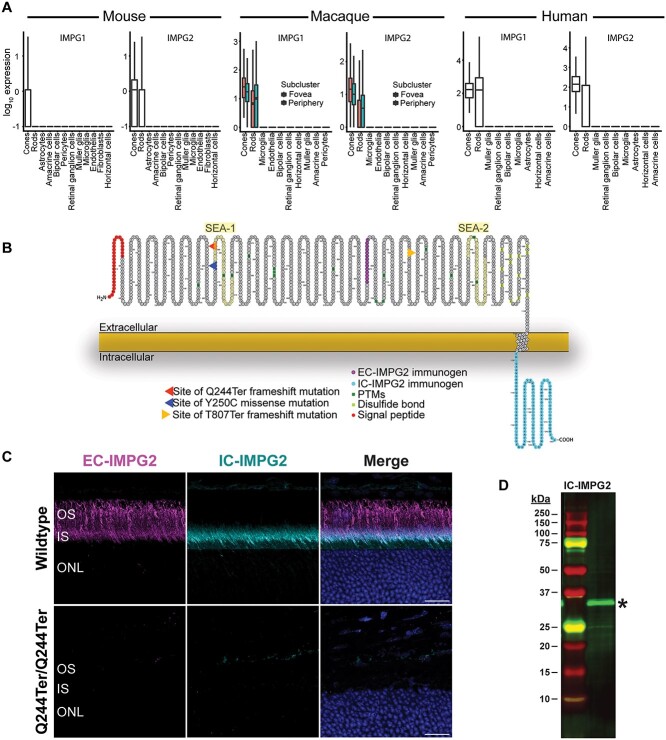
IMPG1 and IMPG2 are selectively localized to photoreceptors. (A) Single-cell RNAseq analysis showing *IMPG1* and *IMPG2* transcript levels in retinal cell types from postnatal day 14 mouse (left), 3–9-year-old rhesus macaque (middle), and 42–80-year-old humans (right). Gene expression is represented on the y-axis as log_10_(x + 0.1), where ‘x’ represents relative transcript levels. IMPG1 and IMPG2 are selectively localized to photoreceptors. See Methods for analyzed datasets. (B) Schematic of mouse IMPG2 highlighting extracellular (EC-IMPG2) and intracellular (IC-IMPG2) epitopes targeted for generation of novel IMPG2 antibodies used in this study. Post-translational modifications (dark green), disulfide bonds (light green), and signal peptide (red) are shown, as are the sites of the Q244Ter frameshift, Y250C missense, and T807Ter frameshift mutations engineered into the mouse models used in this study. The two SEA domains (SEA-1 and SEA-2) are also shown, with proteolysis of the full-length IMPG2 protein expected in SEA-2 based on the conserved ‘GSIVV’ cleavage sequence [53]. (C) Validation of IMPG2 antibodies showing localization of EC-IMPG2 (magenta) in the distal portion of inner segment (IS) and outer segment (OS) of wildtype mice, selective localization of IC-IMPG2 (cyan) in the photoreceptor IS, and an absence of labeling in *Impg2^Q244Ter/Q244Ter^* mice lacking IMPG2. ONL = outer nuclear layer. Images were acquired from peripheral retina. Scale bar, 20 μm. (D) Representative western blot from WT retinal lysate demonstrating that the IC-IMPG2 antibody detects a ~ 33 kDa truncated fragment of IMPG2 (asterisk), consistent with its cleavage in the second SEA domain (SEA-2).

To better understand how *IMPG2* mutations are linked to retinal pathology and vision loss, we first used available single-cell RNA-seq data sets from mouse, macaque, and human retina to examine the expression profiles of *IMPG1* and *IMPG2* in a subset of retinal cell types [35–37]. We found that *IMPG1* is expressed at similarly high levels in both rod and cone photoreceptors in macaque and human. At least in the P14 retina, *Impg1* mRNA in mouse is preferentially expressed in cone photoreceptors (Fig. 1A), but in older mice, whatever the *Impg1* mRNA expression levels may be, the expression levels are clearly sufficient to produce ample IMPG1 protein [38, 39]. *IMPG2* mRNA is expressed at high levels in both rod and cone photoreceptors in mouse, macaque, and human (Fig. 1A). IMPG2 protein consists of a large extracellular domain containing a signaling peptide and an intracellular transmembrane-spanning region (Fig. 1B). Several *IMPG2* disease-causing mutations have been identified, including mutations for which we generated mouse equivalent mutations (Y250C missense mutation or T807Ter frameshift mutation) investigated in this study, along with a mutation (Q244Ter mutation) predicted to fully prevent IMPG2 protein expression in mice (Fig. 1B).

**Figure 2:**
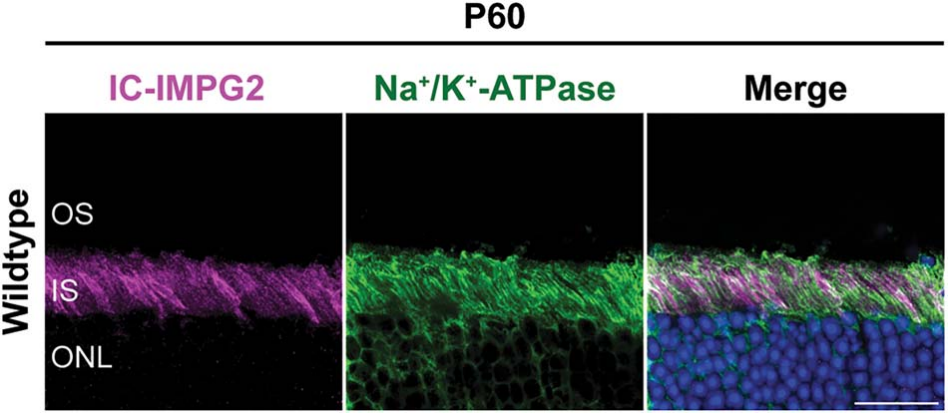
The C-terminal IMPG2 fragment can be observed across IS regions demarcated by Na^+^/K^+^-ATPase labeling. Confocal micrographs showing immunofluorescent labeling of the IMPG2 intracellular fragment (magenta) and Na^+^/K^+^-ATPase in a retinal section from a wildtype mouse. DAPI was used as a nuclear counterstain (blue). Na^+^/K^+^-ATPase labels the full extent of the photoreceptor inner segment (IS) plasma membrane. OS = outer segment, ONL = outer nuclear layer. Scale bar, 20 μm.

To determine if the mutations in our *Impg2* mouse models differentially impact protein expression and lead to pathology associated with IRDs, we generated two novel antibodies to specifically immunolabel either the intracellular (IC-IMPG2) or extracellular (EC-IMPG2) domain of IMPG2 (see Fig. 1B for antibody immunogen sites). In wildtype (WT) mice, EC-IMPG2 immunofluorescence was found prominently surrounding the photoreceptor OS and in the outer portion of the IS, whereas IC-IMPG2 immunofluorescence was isolated to the photoreceptor IS (Fig. 1C). The difference in localization between the extracellular and intracellular domains of IMPG2 suggests the protein may be cleaved [39]. Indeed, the IC-IMPG2 antibody only recognizes an ~33 kDa fragment consistent with complete proteolysis of IMPG2 at the second SEA domain (Fig. 1D). EC-IMPG2 and IC-IMPG2 immunofluorescence was not detected at appreciable levels in *Impg2^Q244Ter/Q244Ter^* mice, thus demonstrating the specificity of our antibodies (Fig. 1C). Immunoreactivity against IC-IMPG2 sometimes appeared greater in the apical region of the photoreceptor inner segment than the basal region (Fig. 1C). However, this enrichment pattern has been observed inconsistently across previous studies [16, 38, 40–45]. We therefore immunolabeled WT retina for both IC-IMPG2 and Na^+^/K^+^-ATPase, which labels the full extent of the inner segment plasma membrane (Fig. 2). No evidence of an IC-IMPG2 gradient at the inner segment was apparent by confocal microscopy, although we cannot rule out the possibility of subtle regional differences of IMPG2 IS localization across the retina.

**Figure 3:**
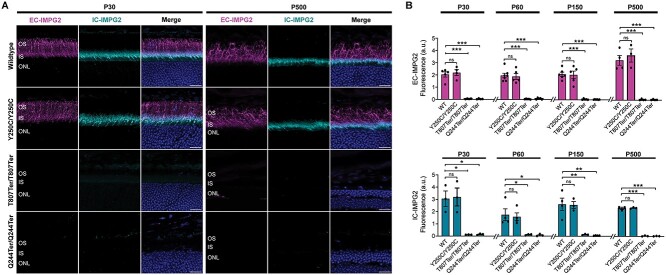
Patient-modeling *Impg2* mutations differentially alter IMPG2 protein expression in mouse retina. (A) Confocal micrographs showing immunofluorescent labeling of the IMPG2 extracellular epitope (EC-IMPG2; magenta), intracellular epitope (IC-IMPG2; cyan), and DAPI (labels nuclei; blue) in the peripheral retina of P30 and P500 WT, *Impg2^Y250C/Y250C^*, *Impg2^T807Ter/T807Ter^*, and *Impg2^Q244Ter/Q244Ter^* mice. In WT and *Impg2^Y250C/Y250C^* mice, EC-IMPG2 stained the complete OS and the outer part of the IS. IC-IMPG2 stained the IS. IMPG2 immunofluorescent was absent in *Impg2^T807Ter/T807Ter^* and *Impg2^Q244Ter/Q244Ter^* mice. (B) Quantitative analysis of integrated fluorescence intensity of EC-IMPG2 and IC-IMPG2 labeling in peripheral retina. There was no detectable IMPG2 in *Impg2^T807Ter/T807Ter^* or *Impg2^Q244Ter/Q244Ter^* mice. Data represent means ± SEM, with scatterplots showing data from separate mice. *p* values were determined by one-way ANOVA followed by Dunnett’s multiple comparison post-hoc test. ^*^p < 0.05, ^*^^*^p < 0.001, ^*^^*^^*^p < 0.0001. Scale bars, 20 μm.

We next investigated the impact of *Impg2* mutations on protein expression in mouse retina via immunocytochemistry (ICC) analyses. Like in WT mice, EC-IMPG2 immunofluorescence in *Impg2^Y250C/Y250C^* mice was mainly detected in the photoreceptor outer segment (OS) with some staining at the distal region of the IS, and IC-IMPG2 was detected in the photoreceptor IS; this pattern of IMPG2 labeling was observed in all the studied timepoints, from P30 till P500 (Fig. 3; Supplementary Material, Fig. S1). In contrast, IMPG2 immunofluorescence was absent in *Impg2^T807Ter/T807Ter^* and *Impg2^Q244Ter/Q244Ter^* mice as early as P30 (Fig. 3; Supplementary Material, Fig. S1). Across postnatal maturation, levels of EC-IMPG2 and IC-IMPG2 remained similar between WT and *Impg2^Y250C/Y250C^* mice, whereas no significant expression of either antigen was observed in *Impg2^T807Ter/T807Ter^* and *Impg2^Q244Ter/Q244Ter^* mice at any age (Fig. 3B).

We studied the relationship between IMPG1 and IMPG2 labeling in (1) WT mice, (2) the different *Impg2* mutation mouse models, and (3) *Impg1* KO mice. At P60, both WT and *Impg2*^Y250C/Y250C^ mice showed IMPG1 and EC-IMPG2 colocalization at the photoreceptor OS level (Supplementary Material, Fig. S2A). Consistent with previous studies in another line of *Impg2* KO mice [38], the loss of IMPG2 protein in *Impg2^T807Ter/T807Ter^* and *Impg2^Q244Ter/Q244Ter^* mice was also linked to mislocalization of IMPG1 protein. These mice exhibited unorganized and discrete presence of IMPG1, with an apparent accumulation of this protein at the OS and RPE boundary (Supplementary Material, Fig. S2A). *Impg1* KO mice lacked IMPG1 antibody labeling and showed diminished EC-IMPG2 staining, with the EC-IMPG2 and IC-IMPG2 labeling largely co-localizing at the IS and innermost part of the OS, similar to previous findings [38] (Supplementary Material, Fig. S2B). These data confirm an inter-dependency between IMPG1 and IMPG2 localization [38].

**Figure 4:**
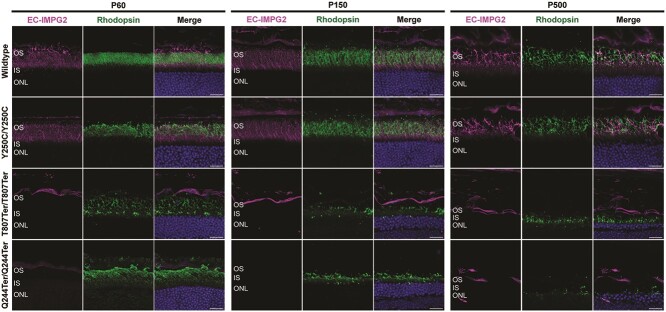
Effects of IMPG2 mutations on rhodopsin expression. Immunofluorescent labeling of the IMPG2 extracellular epitope (EC-IMPG2; magenta), rhodopsin (green), and DAPI (blue) in the peripheral retina of WT, *Impg2^Y250C/Y250C^*, *Impg2^T807Ter/T807Ter^*, and *Impg2^Q244Ter/Q244Ter^* mice at P60, P150, and P500. Images were acquired from peripheral retinas. WT and *Impg2^Y250C/Y250C^* mice showed overlap of rhodopsin and EC-IMPG2 at the rod photoreceptor outer segment. *Impg2^T807Ter/T807Ter^* and *Impg2^Q244Ter/Q244Ter^* mice showed diminished rhodopsin staining. OS = outer segment; IS = inner segment; ONL = outer nuclear layer. Scale bar, 20 μm.

We examined rhodopsin expression to study the impact of *Impg2* mutations on rod photoreceptors. We stained our *Impg2* mouse models across multiple ages using rhodopsin and EC-IMPG2 antibodies. We found strong overlap between rhodopsin and EC-IMPG2 staining of rod OS in WT and *Impg2^Y250C/Y250C^* mice at all timepoints (Fig. 4). *Impg2^T807Ter/T807Ter^* and *Impg2^Q244Ter/Q244Ter^* mice at P60 showed misaligned photoreceptor OS with no EC-IMPG2 expression. Both frameshift mutations resulted in a dramatic diminution of rhodopsin expression with age, with there being a particularly large loss of rhodopsin immunoreactivity in both frameshift mutation mouse models by P500.

**Figure 5:**
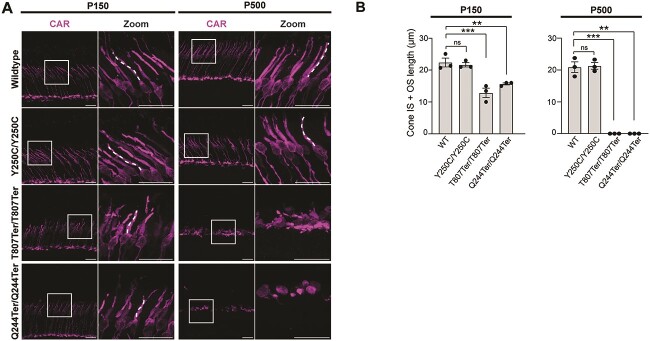
Age-dependent loss of cone photoreceptor integrity in *Impg2^T807Ter/T807Ter^* and *Impg2^Q244Ter/Q244Ter^* mice. (A) Immunofluorescent labeling of the cone arrestin (CAR, magenta) in the peripheral retina of WT, *Impg2^Y250C/Y250C^*, *Impg2^T807Ter/T807Ter^*, and *Impg2^Q244Ter/Q244Ter^* mice at P150 and P500. Cones in both WT and *Impg2^Y250C/Y250C^* mice showed a normal morphology. Changes in cone photoreceptor morphology were apparent in both *Impg2^T807Ter/T807Ter^* and *Impg2^Q244Ter/Q244Ter^* mice. Inset locations are approximate. OS = outer segment; IS = inner segment; ONL = outer nuclear layer. Scale bar, 20 μm. (B) Quantification of photoreceptor (PR) length (mean ± SEM).

We studied the morphology and length of cone OS and IS using a cone arrestin antibody (Fig. 5). In the peripheral retina of WT and *Impg2^Y250C/Y250C^* mice, cone inner and outer segments exhibited normal morphology and were well-aligned. However, at P150, remarkable changes in cone morphology of *Impg2^T807Ter/T807Ter^* and *Impg2^Q244Ter/Q244Ter^* mice were apparent. The cones developed a disorganized distribution and a shorter, swollen IS, although their OS remained aligned. At P500, there was a qualitative reduction in total cone number in *Impg2^Q244Ter/Q244Ter^* and *Impg2^T807Ter/T807Ter^* mice. The extant cones lost their alignment and showed no distinguishable IS or OS (Fig. 5A). We examined putative anatomical difference across the retina and found that cone morphology changed between the peripheral and mid-peripheral retina. At P500, mid-peripheral cones in both *Impg2^T807Ter/T807Ter^* and *Impg2^Q244Ter/Q244Ter^* mice were better preserved, although shorter from pedicle to OS and bearing swollen inner and outer segments (Supplementary Material, Fig. S3). Cone inner and outer segment length was similar between WT and *Impg2^Y250C/Y250C^* mice, and their segment lengths were significantly longer than those in *Impg2^T807Ter/T807Ter^* and *Impg2^Q244Ter/Q244Ter^* mice, in which no cone photoreceptor inner and outer segments could be measured by P500 (Fig. 5B). Arrestin-labeled cones indicated no difference between the number of cones present in the retinas of WT versus *Impg2^Y250C/Y250C^* mice (Supplementary Material, Fig. S4). In the peripheral retina, where cone degeneration was most severe in the two knockout mouse models, WT mice had 20 ± 1.1 cones and *Impg2^Y250C/Y250C^* mice had 19.5 ± 1.7 cones per 100 μm of sectioned retina (*P* = 0.80, n = 3 mice/genotype).

**Figure 6:**
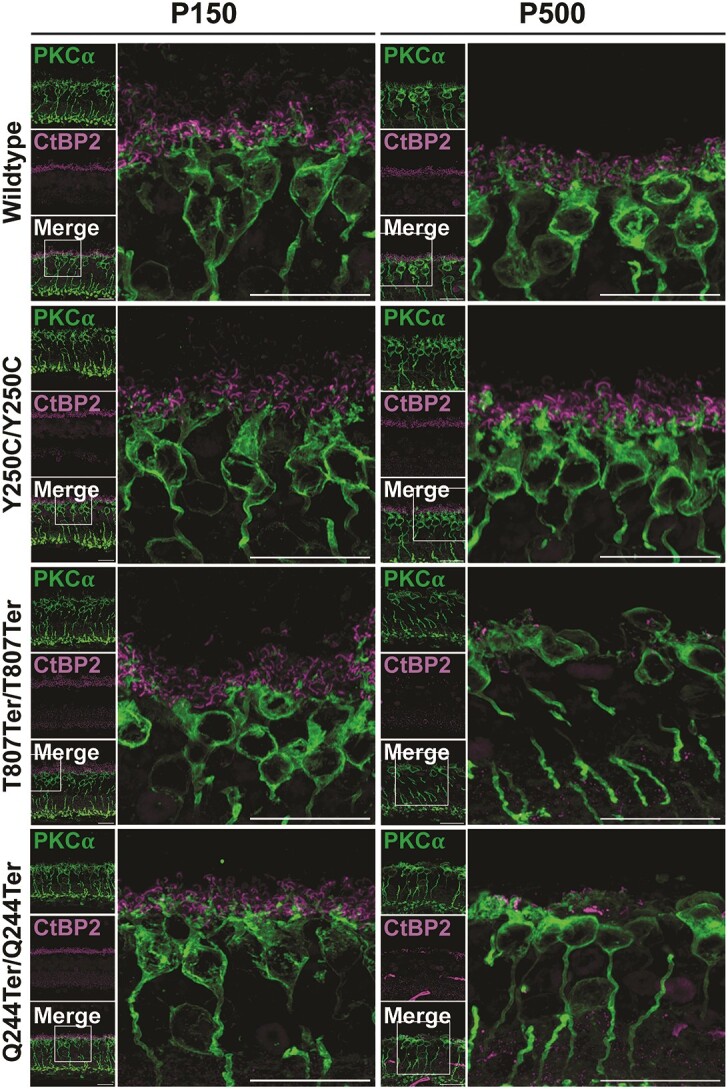
Evidence for loss of ribbon synapses in the retina of *Impg2^T807Ter/T807Ter^* and *Impg2^Q244Ter/Q244Ter^* mice at P150 and P500. Immunofluorescent labeling of PKCα (green, labels rod bipolar cells) and CtBP2 (magenta, labels ribbon synapses) in the peripheral retina of WT, *Impg2^Y250C/Y250C^*, *Impg2^T807Ter/T807Ter^*, and *Impg2^Q244Ter/Q244Ter^* mice at P150 and P500. There was a loss of ribbon synapses in the retina of *Impg2^T807Ter/T807Ter^* and *Impg2^Q244Ter/Q244Ter^* mice at P150 and P500 with disappearance of rod bipolar dendrites at P500. Inset locations are approximate. OS = outer segment; IS = inner segment; ONL = outer nuclear layer. Scale bar, 20 μm.

To check the preservation of the retinal circuitry, we studied the outer plexiform layer (OPL). OPL synapses were identified using an antibody against CtBP2, a marker for synaptic ribbons, and an antibody against protein kinase C (PKC)-alpha, a marker for rod bipolar cells (Fig. 6). In WT and *Impg2^Y250C/Y250C^* mice, the dendritic tips of rod bipolar cells (labeled with PKC-alpha) were associated with ribbon synapses (labeled with CtBP2). Rod bipolar cells in *Impg2^T807Ter/T807Ter^* and *Impg2^Q244Ter/Q244Ter^* mice showed a reduction of their dendrites, with less complex branching patterns and a near absence of dendrites by P500 (Fig. 6). Immunoreactivity for CtBP2 was almost undetectable in older *Impg2^T807Ter/T807Ter^* and *Impg2^Q244Ter/Q244Ter^* mice, and it remained associated with small bipolar dendrites. We also found differences between peripheral and mid-peripheral synaptic ribbons, with the peripheral rod bipolar cells exhibiting a profound disappearance of their dendritic tips and no ribbon synapses, while the mid-peripheral rod bipolar cells showed some dendritic sprouting in contact with the preserved ribbon synapses (Supplementary Material, Fig. S5).

**Figure 7:**
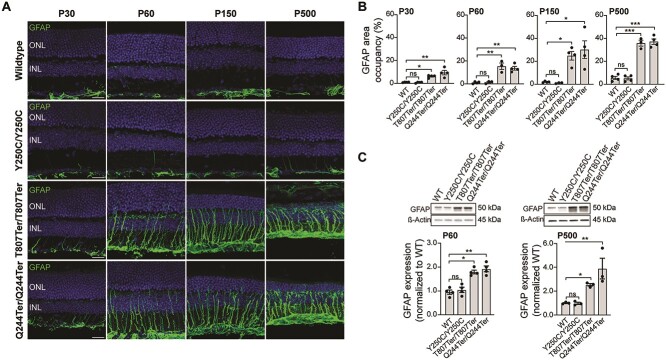
Progressive gliosis in the retina of *Impg2^T807Ter/T807Ter^* and *Impg2^Q244Ter/Q244Ter^* mice. (A) Immunolabeling of GFAP (labels reactive glia; green) in the peripheral retina of WT, *Impg2^Y250C/Y250C^*, *Impg2^T807Ter/T807Ter^*, and *Impg2^Q244Ter/Q244Ter^* mice at P30, P60, P150, and P500. Gliosis was apparent in *Impg^T807Ter/T807ter^* and *Impg2^Q244Ter/Q244Ter^* mice by P30 and increased through P500. OS = outer segment; IS = inner segment; ONL = outer nuclear layer. Scale bar, 20 μm. (B) Quantification (mean ± SEM) of GFAP labeling. Scatter plots represent data from individual mice. The area occupied by GFAP was significantly higher in *Impg2^T807Ter/T807ter^* and *Impg2^Q244Ter/Q244Ter^* than in WT and *Impg2^Y250C/Y250C^* mice. (C) Representative western blots (top) from WT and *Impg2* mutant mice at P60 (left) and P500 (right), demonstrating enhanced GFAP expression in *Impg2^T807Ter/T807ter^* and *Impg2^Q244Ter/Q244Ter^* mice. Graphs represent mean ± SEM, and scatter plots represent data from individual mice. *p* values were determined by one-way ANOVA followed by Dunnett’s multiple comparison post-hoc test. ^*^p < 0.05, ^*^^*^p < 0.001, ^*^^*^^*^p < 0.0001, ns = non-significant.

Gliosis has been previously observed in *Impg2* knockout mice and in other animal models in response to retinal damage [38, 45]. We immunostained and quantified Müller cell reactivity in the different *Impg2* mouse models using glial fibrillary acid protein (GFAP) as a marker. WT and *Impg2^Y250C/Y250C^* mice exhibited similar GFAP expression during all examined postnatal time points (Fig. 7A). In contrast, the *Impg2^T807Ter/T807Ter^* and *Impg2^Q244Ter/Q244Ter^* mice demonstrated a clear gliosis throughout the retina by P30, with a marked GFAP upregulation in Müller cells that persisted from P60 until P500, when a qualitative reduction in total retinal thickness was readily apparent. Statistically significant differences between the WT and *Impg2^Y250C/Y250C^* mice compared to the *Impg2^T807Ter/T807Ter^* and *Impg2^Q244Ter/Q244Ter^* mice were readily apparent by P30 and maintained through P500, the latest age examined (Fig. 7B). These differences were also observed by quantitative western blotting measured at P60 and P500 (Fig. 7C).

**Figure 8:**
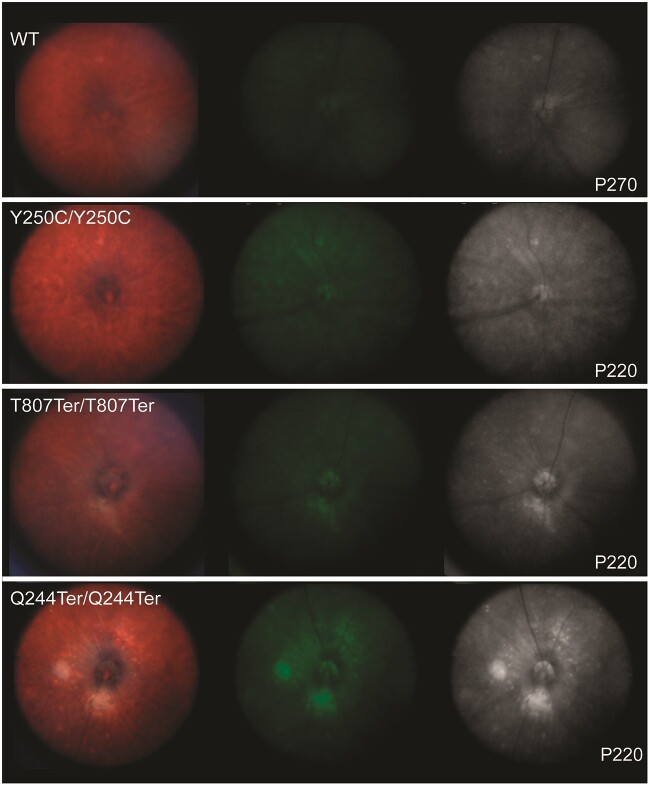
Fundus imaging reveals subretinal deposits in *Impg2^Q244Ter/Q244Ter^* and *Impg2^T807Ter/T807Ter^* mice. Raw fundus images, fundus images with green filter, and green-filtered fundus images converted to grayscale in WT, *Impg2^Y250C/Y250C^*, *Impg2^T807Ter/T807Ter^*, and *Impg2^Q244Ter/Q244Ter^* mice at P220-P270. *Impg2^T807Ter/T807Ter^* and *Impg2^Q244Ter/Q244Ter^* mice exhibit a pale area around the optic disc and subretinal lesions close to the optic disc. WT and *Impg2^Y250C/Y250C^* mice show no defined subretinal deposits, although a drusen-like deposit is apparent in the superior retina of the *Impg2^Y250C/Y250C^* mouse.

In P220-P270 WT and *Impg2* mutant mice, fundus images were acquired with and without a green filter, and the green-filtered images were converted to grayscale to improve visualization of autofluorescence (Fig. 8). A normal RPE could be appreciated in the fundus images from WT mice, and no hyperfluorescence or retinal lesions were apparent. The *Impg2^Y250C/Y250C^* mice showed some RPE changes with no clear lesions in the posterior pole, although small drusen-like deposits were occasionally found. Fundus imaging of *Impg2^Q244Ter/Q244Ter^* and *Impg2^T807Ter/T807Ter^* mice consistently revealed a whitish ring surrounding the optic disc and white, well-defined lesions located deep in the retina and close to the optic disc. These lesions appeared autofluorescent with the green filter and the grayscale conversion, and they reached diameters similar to that of the optic disc.

**Figure 9:**
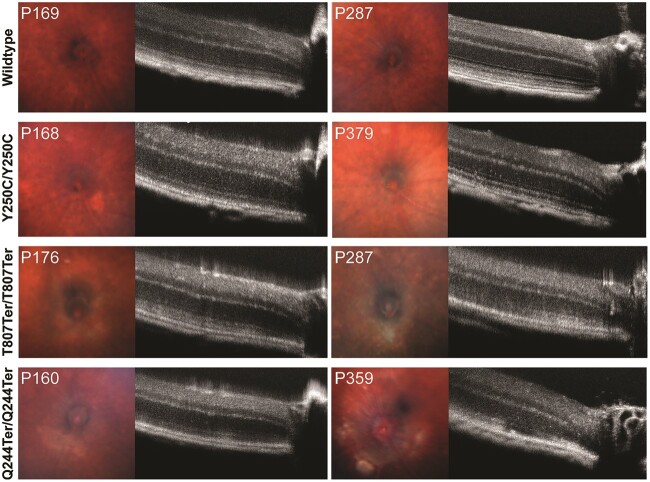
Subretinal deposit formation progressively increases in *Impg2^Q244Ter/Q244Ter^* and *Impg2^T807Ter/T807Ter^* mice. Fundus and OCT imaging in WT, *Impg2^Y250C/Y250C^*, *Impg2^T807Ter/T807Ter^*, and *Impg2^Q244Ter/Q244Ter^* mice at ~P170 and ~1 year of age, as indicated. Fundus imaging reveals progressive growth of white lesions close to the optic disc in both *Impg2^Q244Ter/Q244Ter^* and *Impg2^T807Ter/T807Ter^* mice, and OCT reveals changes in the outer retinal layers, with disruptions and disappearance of the ellipsoid zone and photoreceptor outer segment—RPE interdigitation line.

**Figure 10:**
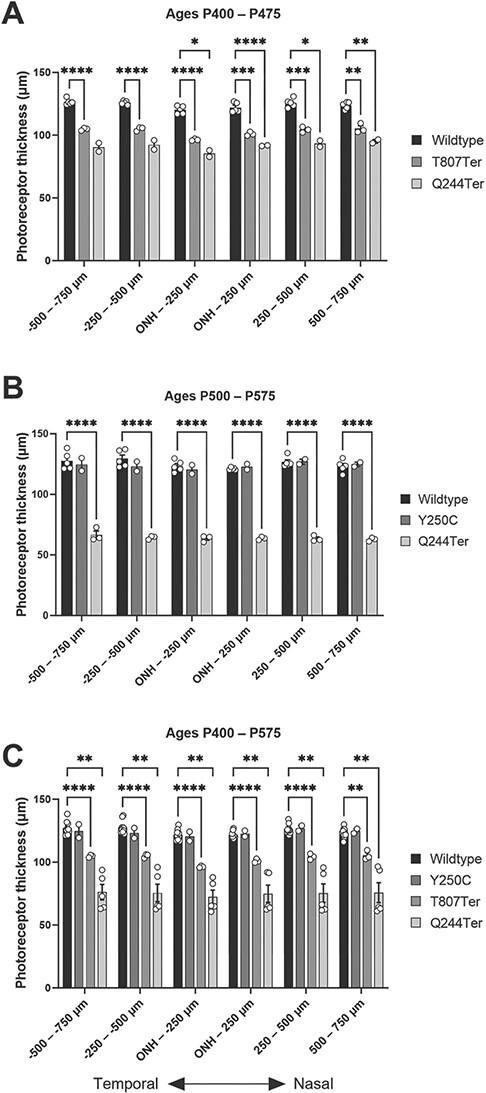
OCT imaging demonstrates that T807Ter and Q244Ter frameshift mutations cause decreased photoreceptor layer thickness. (A) Measurements of photoreceptor layer thickness in P400–475 *Impg2^T807Ter/T807Ter^* (n = 3) and *Impg2^Q244Ter/Q244Ter^* (n = 2) mice are 17% and 26% lower than those of wildtype (n = 5) mice, respectively. (B) Measurements of photoreceptor layer thickness in P500–575 *Impg2^Q244Ter/Q244Ter^* (n = 3) mice are 49% lower than those of wildtype mice, whereas the measurement did not differ between *Impg2^Y250C/Y250C^* (n = 2) and wildtype mice (n = 5). (C) Combined analysis of measurements presented in panels A and B. ONH = optic nerve head. Negative values on the x-axis denote the distance from the border of the optic nerve head in the temporal retina and positive values denote the distance from the border of the optic nerve head in the nasal retina. Error bars represent the SEM. Statistical significance is represented by ^*^p < 0.05, ^*^^*^p < 0.01, ^*^^*^^*^p < 0.001, ^*^^*^^*^^*^p < 0.0001.

A normal OCT profile was observed in the WT mice at two adult ages. The outer retinal hyperreflective lines were visible, including those corresponding to the external limiting membrane (ELM), the ellipsoid zone (EZ), the interdigitation zone (IZ), and the RPE (Fig. 9). *Impg2^Y250C/Y250C^* mice also showed well-defined outer retinal layers with no disruptions. However, *Impg2^Q244Ter/Q244Ter^* and *Impg2^T807Ter/T807Ter^* mice exhibited changes in the outer retina, with hyperreflective material located between the photoreceptor and the RPE layer at the EZ and IZ levels (Fig. 9). In *Impg2^Q244Ter/Q244Ter^* and *Impg2^T807Ter/T807Ter^* mice, the outer hyperreflective lines showed disruptions at the older ages (Fig. 9), with occasional neurosensorial detachment (not shown); the EZ and IZ lost their linear and continuous appearance. Photoreceptor layer thickness was attenuated in P400–P475 mice harboring frameshift mutations in *Impg2*, as measurements were ~15% lower in *Impg2^T807Ter/T807Ter^* mice and ~25% lower in *Impg2^Q244Ter/Q244Ter^* than in WT mice (Fig. 10A)*.* By P500–P575, photoreceptor layer thickness in the *Impg2^Q244Ter/Q244Ter^* mice was reduced to ~50% of that observed in WT mice, whereas measurements collected in mice harboring the *Impg2^Y250C/Y250C^* missense mutation were no different than WT (Fig. 10B). Meaningful differences were not apparent for these effects across the six nasal-temporal regions of retina evaluated (Fig. 10A-C).

**Figure 11:**
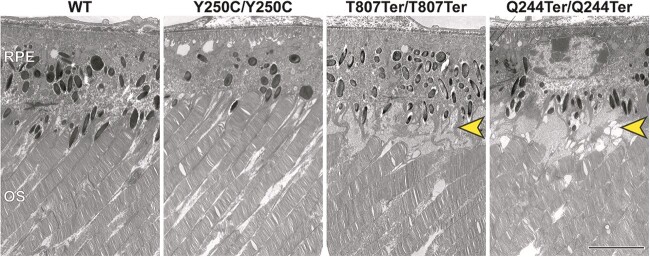
Ultrastructural analysis of subretinal deposits in *Impg2^Q244Ter/Q244Ter^* and *Impg2^T807Ter/T807Ter^* mice. Transmission electron microscopy (TEM) in the outer retina of P90 WT, *Impg2^Y250C/Y250C^*, *Impg2^T807Ter/T807Ter^*, and *Impg2^Q244Ter/Q244Ter^* mice. Extracellular matrix material (yellow arrowhead) accumulates specifically between the outer segments (OS) and the RPE in *Impg2^T807Ter/T807Ter^* and *Impg2^Q244Ter/Q244Ter^* mice. Scale bar, 5 μm.

To gain insights into the earlier manifestations that underlie the formation of retinal deposits, we performed transmission electron microscopy (TEM) at the interface of the photoreceptor outer segments and the RPE in retinas of P90 WT, *Impg2^Y250C/Y250C^*, *Impg2^T807Ter/T807Ter^*, and *Impg2^Q244Ter/Q244Ter^* mice (Fig. 11). We observed an accumulation of extracellular matrix material between the OS and RPE in the *Impg2^T807Ter/T807Ter^* and *Impg2^Q244Ter/Q244Ter^* mice but not in the *Impg2^Y250C/Y250C^* or WT control mice. These observations are consistent with our findings that IMPG1 accumulates at this interface in *Impg2^T807Ter/T807Ter^* and *Impg2^Q244Ter/Q244Ter^* mice (Supplementary Material, Fig. S2A) and that hyperreflective material is detectable at this interface by OCT (Fig. 9).

**Figure 12:**
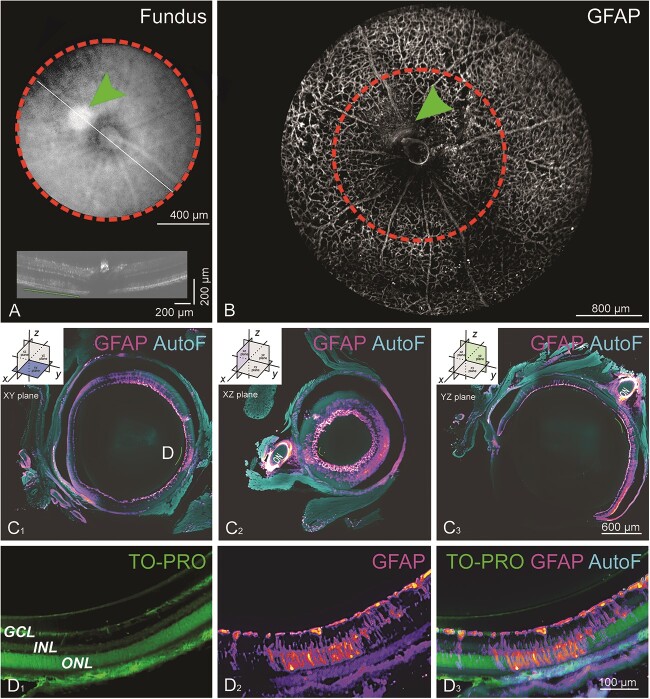
*In vivo* fundus and OCT imaging followed by *ex vivo* light-sheet fluorescence microscopy (LSFM) reveal regions of enhanced gliosis. (A) Fundus photograph and corresponding OCT image in an *Impg2^Q244Ter/Q244Ter^* mouse at P90. The fundus photograph reveals a large white nodule (arrowhead). The cross-sectional OCT image shows the loss of the bands corresponding to the lesion. (B) The same eye was processed for iDISCO clearing. *In silico* dissection of the LSFM dataset shows an *en face* view of the retina stained for GFAP. Blood vessels covered with GFAP-positive astrocytic processes provide readily recognizable landmarks, making it possible to closely match fundus, OCT, and LSFM geography. The arrowhead points to the location of the nodule shown in A. (C) Optical sections of GFAP staining in the (C_1_) xy, (C_2_) xz, and (C_3_) yz planes. A Fire LUT was applied to better depict the GFAP level. Autofluorescence at 488 nm provides anatomical context (light blue). Double arrows mark the location of the hyperreflective nodule in A. This site shows intense GFAP staining of Müller glia extending into the outer retina, in stark contrast with its surrounding, where GFAP staining is less intense and concentrates in the ganglion cell layer. (D) High magnification optical section at the hyperreflective nodule location depicted in C_1_. (D_1_) The nuclear marker TO-PRO-3 allows for the identification of retina layers. (D_2_) GFAP labeling is visible in all retinal layers but concentrate in the outer nuclear layer. (D_3_) Merge image of TO-PRO-3, GFAP, and autofluorescence. GCL = ganglion cell layer; INL = inner nuclear layer; ONL = outer nuclear layer.

Finally, we aimed to link anatomical deficits observed by fundus and OCT to histological markers. For this, we first performed fundus and OCT imaging to identify pathological landmarks (Fig. 12A). We then performed *postmortem* clearing of the eye using iDISCO, immunofluorescence for GFAP and TO-PRO-3 (a nuclear stain), and light-sheet fluorescence microscopy to examine retinal anatomy in three dimensions, which we could optically slice in any desired plane. The GFAP labeling marked astrocytes that decorated the retinal vasculature (Fig. 12B), which we used as landmarks to align the light-sheet fluorescence image to vasculature landmarks observed by fundus imaging. We could then pinpoint subretinal lesions, identified previously by OCT, using immunofluorescent labeling. Particularly high levels of GFAP immunoreactivity were detected at the locations of the subretinal lesions, which is indicative of high levels of reactive Müller glia (Fig. 12C and D).

## Discussion

Here we developed three new *Impg2* mouse models to better reveal the role of IMPG2 in retinal disease and to model patient mutations for preclinical therapeutic development studies: *Impg2^Q244Ter/Q244Ter^* mice were designed to fully prevent the production of IMPG2, *Impg2^Y250C/Y250C^* mice model the Y254C human mutation found in a patient from which pluripotent stem cells were derived and used to generate retinal organoids [34], and *Impg2^T807Ter/T807Ter^* mice mimic the human A805Ter mutation reported in ClinVar and also found in another patient from which pluripotent stem cells were derived and studied [34] (https://www.ncbi.nlm.nih.gov/clinvar/variation/236459/). We found no detectable IMPG2 protein in *Impg2^Q244Ter/Q244Ter^* and *Impg2^T807Ter/T807Ter^* mice, whereas IMPG2 was still expressed in the *Impg2^Y250C/Y250C^* mice. The *Impg2^Q244Ter/Q244Ter^* and *Impg2^T807Ter/T807Ter^* mice both exhibited early onset gliosis in the presence of subretinal deposits, corresponding to the IPM location, and the appearance of subretinal detachment in advanced ages, consistent with other recently developed IMPG2 knockout mouse models [38, 45]. Surprisingly, despite its human equivalent mutation being found in an individual with early onset RP and underlying a profound loss of OS in retinal organoids [34], the presence of homozygous Y250C mutations caused minimal retinal pathology in C57BL/6J mice, even into late adulthood; only some small drusen-like deposits were identified. By mapping the progression of retinal pathology in our three *Impg2* mouse models, we highlight the variability of phenotypic outcomes by mutation type and provide a time course of disease progression in preclinical models to advance upcoming genetic therapies.

Our *Impg2* mouse models failed to exhibit retinal abnormalities associated with advanced RP in humans, such as pigmentary changes, pale optic nerve, or changes in arterial vessels. Instead, the *Impg2^T807Ter/T807Ter^* and *Impg2^Q244Ter/Q244Ter^* mice showed white deposits similar to those in patients with AVMD [33]. The accumulation of vitelliform deposits in humans, like in AVMD, appears in pathological conditions due to the impairment of either photoreceptors or the RPE. The RPE plays critical roles in supporting photoreceptor health through controlling metabolite transport and facilitating OS turnover via daily phagocytosis of the distal OS. It is intriguing to consider how disruption of the interface between photoreceptors and the RPE in *Impg2* knockout mice may contribute to their overall pathology. Yet, given the slower degeneration observed in *Impg2* knockout compared to *Mertk* knockout mice, in which OS phagocytosis by the RPE is prevented [46], the homeostatic relationship between photoreceptors and the RPE is likely to be maintained to some degree in *Impg2* knockout mice despite this disrupted interface. It is possible that the limited lifespan of mice might have prevented our ability to observe retinal pathophysiologies consistent with advanced IRD in our *Impg2* mouse models.

To characterize our *Impg2* mouse models, we felt it was critical to first generate highly specific antibodies that separately target the EC-and IC-IMPG2 domains. Using these antibodies, we observed that the IC-domain of IMPG2 is confined to portions of the IS, while the EC-IMPG2 domain is found in both the IS and OS. This observation mirrors previous observations in human retina [43] and is in line with recent observations in mouse showing that IMPG2 is proteolyzed in its second SEA (sperm protein, enterokinase, and agrin) domain [39]. These data collectively indicate that the cleaved C′-terminal IMPG2 peptide does not translocate past the IS ellipsoid, but that the N′-terminal IMPG2 peptide can translocate across the IPM. Indeed, recent data indicate that IMPG1 is required in the trafficking of the EC-IMPG2 peptide to the OS, and consistent with this, we observed that EC-IMPG2 is restricted to the IS area in the *Impg1* KO mice [38, 39]. Clearly, for the correct localization of IMPG1 and IMPG2 around photoreceptors in the IPM, both proteoglycans need to be expressed. Whether IC-IMPG2 is enriched in the apical portion of the photoreceptor inner segment remains uncertain, and further investigation is needed to resolve whether conflicting results, in the present study and elsewhere, are due to variation in tissue preparation, regional differences across the retina, physiologically induced translocation of IMPG2, or otherwise. Collectively, our observations of differential EC-IMPG2 and IC-IMPG2 expression are intriguing not only from a basic science perspective, but they also have translational implications. For example, it may be sufficient for therapeutic benefit to deliver only the EC-IMPG2 domain in gene replacement strategies. Alternatively, viral delivery of full-length *IMPG2* to only a subset of retinal photoreceptor cells may produce sufficient EC-IMPG2 peptide to distribute across the IPM and treat IMPG2-mediated IRD.

We had hypothesized that the *Impg2^Y250C/Y250C^* mice would exhibit similar retinal defects as mice lacking IMPG2 because (1) the human equivalent mutation (Y254C) produces a protein that largely lacks post-translational modifications thought to be critical for IMPG2’s function, (2) a patient with compound heterozygous A805Ter and Y254C mutations exhibited early (<6 years of age) presentation of RP, and (3) heterozygous gene-corrected (Y254C/+), but not homozygous gene-corrected, human induced pluripotent stem cell retinal organoids from this patient failed to maintain photoreceptor OS [34] (unpublished data). There are several possibilities for the unexpectedly mild consequences of the Y250C mutation in mice. One possibility is that the mutation fails to disrupt posttranslational modifications in mice as the Y254C mutation does in humans [34]. Another possibility is that the role of IMPG2 or its posttranslational modifications may differ in human and mouse; there is some precedent for this given that the posttranslational modifications of IMPG1 differ in mouse and human [47]. Incomplete penetrance and variable expressivity have also been related to IMPG2-mediated IRD [27]. However, the most parsimonious explanation may be that both the mouse Y250C and human Y254C mutations are pathogenic, but not to the extent of complete IMPG2 deletion, such that there is minimal retinal pathology that become manifest over the lifespan of mice. Consistent with this idea, the pathogenic consequences of the Y254C *IMPG2* mutation in humans take time to manifest clinically, and even when paired in a compound heterozygous A805Ter mutation, are generally only observed after several years or more in humans. If cones are more severely impacted by the loss of IMPG2 than rods, then it might also be possible that the absence of a macula (area of high cone density) in the mouse precludes rapid pathology in the *Impg2^Y250C/Y250C^* mice [48, 49]. Finally, we cannot rule out the possibility that the pathogenic consequences of Y254C can only be readily observed on an accelerated time course in the challenging *in vitro* physical environment of retinal organoids [34].

In general, our findings of retinal pathology in the *Impg2^Q244Ter/Q244Ter^* and *Impg2^T807Ter/T807Ter^* mice are in close agreement with other recent *Impg2* knockout mouse models, including observations of early gliosis, subretinal deposits, and photoreceptor degeneration with opsin mislocalization [38, 45]. Previous studies suggest that the peripheral retina may be the initial site of disease progression caused by the loss of IMPG2 [45], and we similarly found marked changes in peripheral cones and bipolar cells compared to those in mid-peripheral retina. In the mid-periphery, we observed that cells were better preserved and the bipolar cells showed dendritic sprouting as if in search for presynaptic contacts, as observed in other models of retinal degeneration [50].

We aimed to characterize the trajectory of retinal pathogenesis in our models to provide outcomes for preclinical studies. Previous studies showed that IMPG2 is not detectable until P5-P8 in rodents [41, 42]; thus, we did not anticipate retinal pathologies to emerge until well after the first postnatal week. We found evidence of gliosis by ~P30 in *Impg2^Q244Ter/Q244Ter^* and *Impg2^T807Ter/T807Ter^* mice, which became more widespread and intense with age. Fundus and OCT imaging revealed the consistent presence of subretinal deposits by P100, albeit on rare occasions we observed subretinal deposits in the *Impg2* frameshift mouse models as young as P60 or failed to observe deposits in mice as old as P100. These vitelliform-like lesions are likely due to an accumulation of material at the IPM-RPE interface, including the accumulation of IMPG1 [38]. In support of this idea, we observed an apparent accumulation of debris in the subretinal space between the OS and RPE in the two *Impg2* frameshift model mice (Fig. 9). By P150, we saw evidence for impaired cone cell elongation, consistent with previous observations at ~6 months of age in *Impg2* knockout mice [45], and by P400, these mice demonstrated substantial losses in photoreceptor layer thickness.

Light-sheet microscopy used in tandem with fundus and OCT imaging demonstrated that the intense gliosis observed in mice with *Impg2* frameshift mutations was localized to subretinal deposits. We suggest that it may be possible to extend this approach to preclinical research in which therapeutics (e.g., gene therapy) are evaluated for the treatment of retinal disease, such as IMPG2-RP. In this way, biodistribution of the test material could be precisely mapped to regional differences in pathology versus rescue, across the entire retina in 3D space.

## Materials and methods

All experimental procedures conformed with the Association for Research in Vision and Ophthalmology (ARVO) Statement for the Use of Animals in Ophthalmic and Vision Research, and were approved by the Institute for Animal Care and Use Committee at the University of North Carolina.

### Generation of *Impg2* mouse models

*Impg2^Q244Ter/Q244Ter^*, *Impg2^Y250C/Y250C^*, and *Impg2^T807Ter/T807Ter^* mice were produced by the UNC Animal Models Core. All mouse strains were generated and maintained on a C57BL/6J background. We utilized CRISPR/Cas9 genome editing to generate each of the mouse strains, which were backcrossed at least 2 generations to WT C57BL/6J mice to remove any unintended off-target effects.

For the Y250C mice, we introduced a point mutation (TAC to TGC) into exon 8 of the mouse *Impg2* gene, causing an amino acid mutation from tyrosine 250 to cysteine, precisely modeling the human IMPG2 Y254C mutation (TAC to TGC). The Y250C allele also included a synonymous mutation in the leucine 245 codon (CTT to TTG) to disrupt Cas9/guide RNA binding and cleavage of the modified allele. For the Y250C mutant mouse, the wild-type DNA sequence 5′- AATTCAGCATCCAA**C**T**T**CTGGGGAAGCGAT**A**CAGTGAAGAAC-3′ was modified into 5′- AATTCAGCATCCAA**T**T**G**CTGGGGAAGCGAT**G**CAGTGAAGAAC-3′.

We produced the Q244Ter mice in the process of generating the Y250C mice, as we generated a mouse with a 7 base pair frameshift deletion in exon 8 in the mouse *Impg2* gene, thus initiating multiple stop codons in exon 11. The sequence of the guide RNA (gRNA) used to produce both Y250C and Q244Ter mice was 5′-GATCGCTTCCCCAGAAGT**TGG**-3′ (bold sequence is the PAM)*.* For the Q244Ter mice, the wild-type DNA sequence 5′-AATTCAGCATC**CAACTTC**TGGGGAAGCGATACAGTGAAGAACT-3′ was mutated with deletion of the 7 bolded bases to give 5′-AATTCAGCATCTGGGGAAGCGATACAGTGAAGAACT-3′. This deletion caused a ribosomal frameshift with premature stop codons in exon 11.

For the T807Ter mice, we introduced a deletion of 2 base pairs (CA) into exon 14 of the mouse *Impg2* gene that changes tyrosine 807 into a premature stop codon, thus modeling a patient Ala805Ter frameshift mutation (TG del → TGA termination codon). The T807Ter mutation also included a synonymous mutation of AGA to AGG in the arginine 809 codon downstream of the T807Ter to facilitate genotyping of the animals. The sequence of the guide RNA (gRNA) for the T807Ter mice was 5′-GAGAGCACTGACAGACTC**TGG**-3′ (bold sequence is the PAM)*.*

For the T807Ter mutant mouse, the wild-type DNA sequence 5′-AAGTACACCAGAGAG**CA**CTGACAGACTCTGGTTGAAAGCTT-3′ was changed into 5′-AAGTACACCAGAGAGCTGACAGGCTCTGGTTGAAAGCTT-3′ by deletion of the bolded nucleotides and insertion of the A to G mutation 8 bp downstream.

We identified founder animals with the correct genotype for each desired mutation and crossed the founder mice with C57BL/6J mice purchased from Jackson Laboratories to establish the colonies. We verified genetic manipulation in these mice by Sanger sequencing as follows: DNA from either a small toe, tail, or ear sample was extracted; PCR products of approximately 500 bp were amplified using forward primer *Impg2*-T807-ScF1 (5′-CCAAACCACCCTTCTTACCG-3′) and reverse primer *Impg2*-T807-ScR1 (5′- AAACCACCAATGCTCCTGC-3′) for *Impg2*-T807Ter mice, *Impg2*-Y250-ScF1 (5′- TGCTCTTCCTTGTCAATGTGC-3′) and reverse primer *Impg2*-Y250-ScR1 (5′- GTTGTTGTTATGAGAGAGTTAGTGCC-3′) for *Impg2*-Y250C-KI mice. For the *Impg2*-Q244Ter-InDel mice, two PCR products of approximately 250 bp were amplified using forward primer *Impg2*-WT-F1 (5′- CGGAATTCAGCATCCAACTTC-3′) and reverse primer *Impg2*-Y250-ScR1 (5′- GTTGTTGTTATGAGAGAGTTAGTGCC-3′) for the WT-specific product band and forward primer *Impg2*-Del7-F1 (5′-GCGGAATTCAGCATCTGG-3′) and reverse primer *Impg2*-Y250-ScR1 (5′-GTTGTTGTTATGAGAGAGTTAGTGCC-3′) for deletion-specific product band. The PCR product was purified using the ISOLATE II PCR and Gel Kit (Bioline) according to the manufacturer’s instructions and the purified product was sequenced by Sanger sequencing (Eton Biosciences) using the nested primer *Impg2*-T807-SqF1 (5′-AGGAAGATATGGTACATACAGAATCATC-3′) and *Impg2*-Y250-SqF1 (5′-TGTGAAACTCTGAGGGGAGTCAG-3′) for *Impg2*-T807Ter and both *Impg2*-Y250C and *Impg2*-Q244Ter. Identification of the 2 bp deletion at base pair 2638 followed by a frameshift for *Impg2*-T807Ter mice, or A to G transition at base pair 968 for *Impg2*-Y250C or the 7 bp deletion at base pair 949 followed by a frameshift for *Impg2*-Q244Ter were examined to confirm homozygosity of the mutant alleles.

### Generation of *Impg1* knockout (KO) mouse model

The *Impg1* KO allele design called for deletion of exon 5, which was predicted to cause a ribosomal frameshift with multiple stop codons to cause nonsense mediated mRNA decay. Cas9 guide RNAs were identified in mouse *Impg1* introns 4 and 5 using Benchling software. The guide RNAs selected for genome editing in embryos were 5g67B (protospacer sequence 5′-CTGTTGTGGACCGAATACAG-3′) and 3g59B (protospacer sequence 5′-TTCAAATCGCTAATTTCTCA-3′). Selected guide RNAs were ordered as Alt-R CRISPR-Cas9 crRNA synthetic RNA oligonucleotides from Integrated DNA Technologies (IDT). Synthetic tracrRNA was also ordered from IDT. A donor oligonucleotide, Impg1-Del5-T (sequence 5′-GGATCTGAAGTCTGACCCCCCCAACTTCCTGGCTGGCTATCACTGTGCACCACTGAAATCGCTAATTTCTCAATAATTTCTGGGGCTAGTAATATAGCACAGTTGAGAGT−3′) was ordered from Sigma and included in embryo microinjection to facilitate homologous recombination to produce a clean deletion event between the guide RNA cut sites.

The crRNA and tracrRNA oligonucleotides were resuspended at 100 μM concentration in microinjection buffer (5 mM Tris-HCl pH 7.5, 0.1 mM EDTA). Guide RNA complexes were formed by mixing each crRNA with tracrRNA at a final concentration of 10 μM each in microinjection buffer, heating to 95°C for 5 min and allowing to cool slowly to room temperature. A microinjection mix was prepared by mixing 400 nM recombinant Cas9 protein (Animal Models Core/UNC Protein Expression Core Facility), 250 nM each crRNA/tracrRNA complex, and 100 ng/μl oligonucleotide. The resulting microinjection mix was injected into C57BL/6J zygotes, which were then implanted in recipient pseudopregnant females. The resulting pups were screened by PCR and sequencing for the presence of the desired deletion allele. Nine of 22 pups born had clear evidence of deletion events by PCR. Sequence analysis of PCR amplicons identified 5 animals with a perfect match to the predicted deletion allele. Other animals analyzed had additional deletions and/or insertions at the cut site junction. Two male founders with the correct deletion were mated to wild-type C57BL/6J females for germline transmission of the deletion allele. One founder transmitted the deletion allele to offspring.

### Bioinformatics

A website containing all code and figures comprising our bioinformatics analyses are available at https://jeremymsimon.github.io/Williams_Retina_scRNA_IMPG2_workflowr/index.html. Each species-level analysis has its own dedicated sub-page with links and instructions for data retrieval and analysis.

###  

Mouse bioinformatics: Single-cell RNA-seq data from the P14 mouse retina corresponding to Macosko *et al.* [36] were retrieved from GEO accession GSE63472. A csv file mapping cell barcodes to clusters was additionally downloaded from the McCarroll lab website (https://mccarrolllab.org/wp-content/uploads/2015/05/retina_clusteridentities.txt). The gene expression matrix was subset just for *Impg1* and *Impg2* expression values, and individual clusters were grouped into broader cell classifications consistent with Fig. 5 of the Macosko study [36]. Data were summarized and plotted in R v4.1.0 and all code for this specific analysis is available on GitHub at: https://jeremymsimon.github.io/Williams_Retina_scRNA_IMPG2_workflowr/Macosko_mouse_retina_summarized_IMPG1-2_expression_boxplots.html.

###  

Macaque bioinformatics: Single-cell RNA-seq data from the 3-9yo macaque retina corresponding to Peng *et al.* [37] were retrieved from the Broad single-cell portal https://singlecell.broadinstitute.org/single_cell/study/SCP212/molecular-specification-of-retinal-cell-types-underlying-central-and-peripheral-vision-in-primates. Gene expression matrices for each cell type were subset just for *IMPG1* and *IMPG2* expression values, and individual clusters were grouped into broader cell classifications consistent with Fig. 1 of the Peng study. Data were summarized and plotted for all cell types of the fovea and periphery in R v4.1.0 and all code for this specific analysis is available on GitHub at: https://jeremymsimon.github.io/Williams_Retina_scRNA_IMPG2_workflowr/Peng_macaque_retina_summarized_IMPG1-2_expression_boxplots.html.

###  

Human bioinformatics: Single-cell RNA-seq data from the adult human retina (aged 42–80 years) corresponding to Lukowski *et al.* [35] were retrieved from ArrayExpress E-MTAB-7316, and a file mapping cell barcodes to clusters was additionally supplied by Sam Lukowski (personal communication in October 2019). The gene expression matrix was subset just for *IMPG1* and *IMPG2* expression values, and the values were plotted for each broader cell classification consistent with Fig. 1 of the Lukowski study [35]. Data were summarized and plotted in R v4.1.0 and all code for this specific analysis is available on GitHub at: https://jeremymsimon.github.io/Williams_Retina_scRNA_IMPG2_workflowr/Lukowski_human_retina_summarized_IMPG1-2_expression_boxplots.html

### Animal husbandry and care

Mice were maintained in the UNC vivarium under a 12:12 light:dark cycle (~50 lx illumination during light cycle; <10 lx illumination during dark cycle). Food and water were available *ad libitum*.

### Histology and immunofluorescence

Mice at postnatal day (P) 30, P60, P150, and P500 were euthanized with a lethal dose of isoflurane followed by cervical dislocation. Eyes were collected and hemisected. Eye cups were then fixed in ice-cold 4% paraformaldehyde for 30 min and washed three times with 0.1 M phosphate buffer (PB) containing 1% glycine. Eye cups were then kept in 30% sucrose at 4°C overnight and frozen in a 1:1 (wt/vol) mixture of Optimal Cutting Temperature compound and 30% sucrose in an isopentane ice bath. Frozen eye cups were cryosectioned (Thermo Scientific CryoStar NX50) at 20 μm, mounted on Fisherbrand colorfrost Plus microscope slides (Fisher Scientific), dried for 10–15 min at room temperature (RT), and stored at −80°C. Slides with mounted retina cryosections were warmed to RT, washed with 0.1 M PB for 30 min, and blocked with 0.1 M PB, 10% goat serum, and 0.5% Triton-X100 for 30 min at RT. All immunolabeling steps were carried out at RT in a humidified chamber. In addition to the employed commercial antibodies (see Table 1), we generated custom antibodies to mouse IMPG2 in collaboration with GenScript (Piscataway, NJ, USA): N-terminal antibodies were generated in both rabbit (polyclonal) and mouse (clone 15F3E11) against a KLH-conjugated CQIIESSEHRYGDRP peptide; C-terminal antibodies were generated in rabbit against a His-tagged peptide comprising amino acids 1128–1243 (Q80XH2). Primary antibodies were diluted in 0.1 M PB, 10% goat serum, and 0.5% Triton-X100 at concentrations specified in Table 1, and secondary antibodies were diluted in 0.1 M PB, 10% goat serum, and 0.5% Triton-X100 at a concentration of 1:500. Retinal sections were incubated with primary antibodies overnight and then washed five times with 0.1 M PB. Sections were incubated with secondary antibodies for 30 min, followed by five 0.1 M PB washes. Slides containing labeled retinal sections were mounted with coverslips (Fisherbrand) using Fluoromount-G (SouthernBiotech) and imaged using Olympus FV3000RS Confocal Microscope with FV31S-SW software. Note that the IMPG2 antibodies showed high specificity, as evidenced by the near lack of labeling in *Impg2^Q244Ter/Q244Ter^* mice, but we occasionally observed non-specific binding at the RPE and sclera (for example, as seen in Fig. 4 and Supplementary Material, Fig. S1). Histological data were analyzed offline with Fiji software. All confocal images presented are maximum z-projections where saturation masks were used to prevent signal saturation. The fluorescence intensity of IMPG2 immunolabeling was measured with ImageJ software, where the extent of the outer and inner segments (OS and IS respectively) was analyzed at 60X magnification across a 106 μm width. Data represent pooled results from at least three animals per genotype (minimum of 3 sections/animal). To measure GFAP percent area occupancy (label above threshold), we measured a 127 μm width at 40× magnification from the inner edge of the inner nuclear layer (INL) to 5 μm above the ganglion cell layer (GCL), and the peripheral retina was measured from at least three animals per genotype (minimum of 3 sections/animal). To measure the length of the cone segments, Imaris software was used. Statistical analysis and preparation of graphs were performed using GraphPad Prism software. Figures were made using GraphPad and/or Adobe Illustrator software.

**Table 1:** Antibodies used in this study.

Antibody	Name	Host	Concentration	Source
IMPG2	EC-IMPG2	Rabbit	1:1000	GenScript (customized)
IMPG2	EC-IMPG2	Mouse	1:1000	GenScript (customized)
IMPG2	IC-IMPG2	Rabbit	1:1000	GenScript (customized)
IMPG1	SPARC	Mouse	1:1000	Santa Cruz Biotechnology
GFAP	Glial Fibrillary Acidic Protein	Rabbit	1:800–1:1000	Agilent
Cone Arrestin	Anti-Cone Arrestin	Rabbit	1:200	BD Biosciences
CtBP2	C-Terminal Binding Protein-2	Mouse	1:500	BD lab
PKCα	Anti-Protein Kinase Cα	Rabbit	1:2000	Sigma
Rhodopsin	Anti-Rhodopsin (4D2)	Mouse	1:3000	ABCAM
Na^+^/K^+^-ATPase	ATP1A1	Mouse	1:500	ThermoFisher

### Western blotting

Retinas were dissected from eyes collected from P60 and P500 mice and homogenized by sonication in RIPA buffer (50 mM Tris-HCI pH 8.0, 150 mM NaCl, 1% Nonidet P-40, and 0.5% Sodium Deoxycholate) containing 0.5% SDS and protease inhibitor (Sigma P8340). After centrifugation at full speed for 10 min at 4°C, supernatants were collected, and protein concentrations were measured with the Pierce bicinchoninic acid (BCA) protein assay kit. For GFAP western blotting, 20 μg of retinal protein was mixed with 4× Protein Loading Buffer (Li-Cor), and 10% β-mercaptoethanol, boiled at 95°C for 10 min, and then centrifuged for 1 min at full speed. The prepared protein samples were separated in a 4%–20% SDS–polyacrylamide gel electrophoresis and transferred to polyvinylidene fluoride membranes in transfer buffer (25 mM Tris-base, 192 mM glycine, and 20% MeOH). The membranes were then blocked in Intercept Blocking Buffer for 1 h at RT. Membranes were then probed with primary antibodies overnight at 4°C and then secondary HRP-conjugated antibodies for 1 h at RT. Chemiluminescence reaction was performed using Clarity Western ECL Substrate or SuperSignal West Atto Ultimate Sensitivity Chemiluminescent Substrate. The reaction was imaged by an Amersham Imager 680 (GE Healthcare). Data were analyzed offline with ImageJ software. Western blot signals of GFAP were standardized to that of β-actin, and these standardized data were normalized to the average level in WT mice. Statistical analysis and preparation of graphs were performed using GraphPad Prism software. The data were initially analyzed for normality. Data were incorporated into figures using GraphPad and Adobe Illustrator software.

For the western blot in Fig. 1D, retinas were dissected from WT eyes and homogenized by sonication in 2% SDS containing cOmplete protease inhibitor (Roche). After centrifugation at 20 000 × *g* for 10 minutes at RT, supernatants were collected, and protein concentrations were measured with the RC DC Protein Assay kit (Bio-Rad). 10 μg of retinal protein was mixed with 4x Laemmli Sample Buffer (Bio-Rad) and 100 mM DTT, boiled at 95°C for 5 min, and then centrifuged for 1 min at full speed. The prepared protein samples were separated in a 10%–20% SDS-PAGE gel and transferred to polyvinylidene fluoride membranes in transfer buffer (25 mM Tris-base, 192 mM glycine, and 15% MeOH). The membranes were then blocked in Intercept Blocking Buffer for 1 h at RT. Membranes were then probed with rabbit anti-IC-IMPG2 overnight at 4°C and then secondary IRDye 800CW donkey anti-rabbit IgG for 2 h at RT. Probed blot was imaged by a Li-Cor Odyssey CLx. Data were analyzed offline with ImageJ software.

### Fundus and OCT imaging

Fundus and spectral domain OCT images were acquired in P160–P575 mice using the Micron® IV ophthalmic imaging system (Phoenix Research Labs, Pleasanton, CA). Mice were anesthetized using an injected (i.p.) mixture of ketamine (90 mg/kg) and xylazine (10 mg/kg), and a homeothermic blanket was used to maintain a constant 37°C body temperature. Eyes were dilated with topical application of Phenylephrine (1%) and Tropicamide (1%). Optixcare Eye Lube Plus (CLCMEDICA, Ontario, Canada) was applied to the cornea to prevent dehydration and to improve image quality. Bright field and green filtered fundus images were collected with the optic disc at the center of the field. Single line scan OCT images were acquired in the axial plane at approximately the level of the optic nerve head, providing a view of temporal and nasal retina. In mice P400 and older, OCT images were segmented using InSight 2D software (Voxeleron, Austin, TX) according to the manufacturer’s instructions, and the thickness of the photoreceptor layer was measured as the distance between the outer plexiform layer and the interface of the RPE and choroid. InSight provided values at intervals of ~2 μm across a 2090 μm field of view. A custom Python (Python Software Foundation, Wilmington, DE) script averaged measurements of photoreceptor layer thickness at ~ 250 μm intervals across the retina from the border of the optic nerve head, up to 750 μm. All images had at least 67 μm (33 measurements) in the outermost regions (i.e. −500 μm to −750 μm, 500 μm to 750 μm). Sample sizes for mice P400-P575 (n = total, P400–475, P500–575): WT (n = 10, 5, 5), *Impg2^T807Ter/T807Ter^* (n = 3, 3, 0), *Impg2^Y250C/Y250C^* (n = 2, 0, 2), *Impg2^Q244Ter^/^Q244Ter^* (n = 5, 2, 3). Each sample is the average of the fellow eyes, with the exception of three instances in which data were available for only one eye. GraphPad Prism version 10.0.2 (Dotmatics, Boston, MA) was used to compete the statistical analysis on photoreceptor layer thickness in which each mutant mouse line was compared to the WT control line. Formal comparisons between retinal locations were not included. A two-way ANOVA was performed using the Dunnet post-hoc test to correct for multiple comparisons testing.

### Light-sheet microscopy

Whole eyes immunolabeling and clearing were performed following the iDISCO method from Renier *et al.* [51] with slight variations. First, to oxidize endogenous pigments and quench autofluorescence, the eyes were treated with gradually increasing concentrations (0.3%, 3%, and 10%) of H_2_O_2_ in PBS at 55°C, followed by overnight incubation in a freshly prepared 10% H_2_O_2_/PBS solution. The eyes were then dehydrated in a series of 30 min methanol/PBS mixtures (5%, 10%, 20%, 30%, 40%, 50%, 60%, 70%, 80%, 90%, and 100%) and incubated overnight in a 66% dichloromethane and 33% methanol solution. They were then rehydrated in a series of 30 min methanol/PBS mixtures (100%, 90%, 80%, 70%, 60%, 50%, 40%, 30%, 20%, and 10%). After being washed twice for 30 min in 0.2% TritonX-100 in PBS, samples were incubated at 37°C for two days in permeabilization solution (0.2% TritonX-100, 2% glycine, 20% dimethylsulfoxide in PBS) followed by two days in blocking solution (0.2% TritonX-100, 1% bovine serum albumin). The eyes were incubated for three days with chicken GFAP antibody (1:11000; PA1-1004, RRID: AB_1074620, lot: XD3573631, Invitrogen) in antibody solution (0.2% Tween-20, 40 mg/l heparin, 11% bovine serum albumin, 5% dimethylsulfoxide in PBS). Next, the samples were washed (0.2% Tween-20, 40 mg/l heparin in PBS) and incubated in a mixture of Alexa Fluor 790 Donkey Anti-Chicken IgY (1:200; 703-655-155, RRID: AB_2340382, lot:152183, Immunoresearch Jackson) and TO-PRO-3 iodide (1:1000; T3605, ThermoFisher) in antibody solution for three days. Whole eyes were washed, dehydrated as described above, and processed for cleaning. Samples were incubated overnight in 100% methanol, then for 3 hours in 66% DCM/33% MeOH solution, washed twice in 100% DCM, and cleaned in dibenzyl ether. Imaging was performed using a LaVision BioTec Ultramicroscope II. Visualization was performed using Imaris software (Bitplane).

### Transmission electron microscopy (TEM)

Fixation and processing of eyes for TEM was performed following previously published protocols [52]. In short, anesthetized mice were transcardially perfused with 2% paraformaldehyde, 2% glutaraldehyde, and 0.05% calcium chloride in 50 mM MOPS (pH 7.4). Enucleated eyes were post-fixed for 2 h in the same fixative. Eyecups were dissected, embedded in 2.5% low-melt agarose (Precisionary, Greenville, NC) and sectioned on a Vibratome (VT1200S; Leica, Buffalo Grove, IL). Agarose sections were stained with 1% tannic acid (Electron Microscopy Sciences, Hatfield, PA) and 1% uranyl acetate (Electron Microscopy Sciences), gradually dehydrated with ethanol, and infiltrated and embedded in Spurr’s resin (Electron Microscopy Sciences). 70 nm sections were cut onto copper grids and counterstained with 2% uranyl acetate and 3.5% lead citrate (19 314; Ted Pella, Redding, CA). Samples were imaged on a JEM-1400 electron microscope (JEOL, Peabody, MA) at 60 kV with a digital camera (BioSprint; AMT, Woburn, MA). Image analysis and processing was performed with ImageJ.

## Supplementary Material

impg2_supplement_2_clean_ddad199
