# Correction to: Heterogeneity in the progression of retinal pathologies in mice harboring patient mimicking *Impg2* mutations

**DOI:** 10.1093/hmg/ddae004

**Published:** 2024-01-13

This is a correction to: Brittany N Williams, Adam Draper, Patrick F Lang, Tylor R Lewis, Audrey L Smith, Steven J Mayerl, Marie Rougié, Jeremy M Simon, Vadim Y Arshavsky, Scott H Greenwald, David M Gamm, Isabel Pinilla, Benjamin D Philpot, Heterogeneity in the progression of retinal pathologies in mice harboring patient mimicking Impg2 mutations, *Human Molecular Genetics*, 2023; https://doi.org/10.1093/hmg/ddad199

In the originally published version of this manuscript there were errors within Figure 10 and its legend. Figure 10 should read:


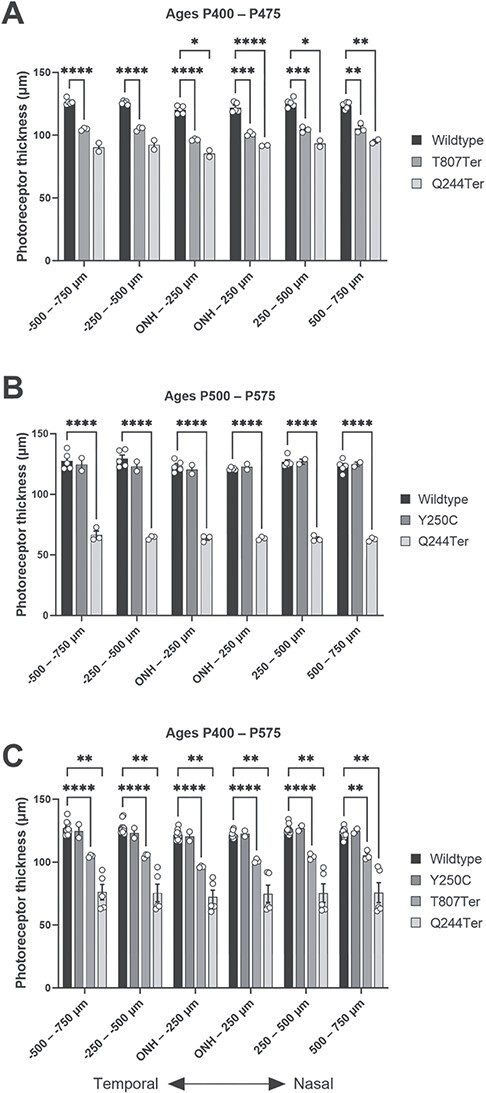


instead of:


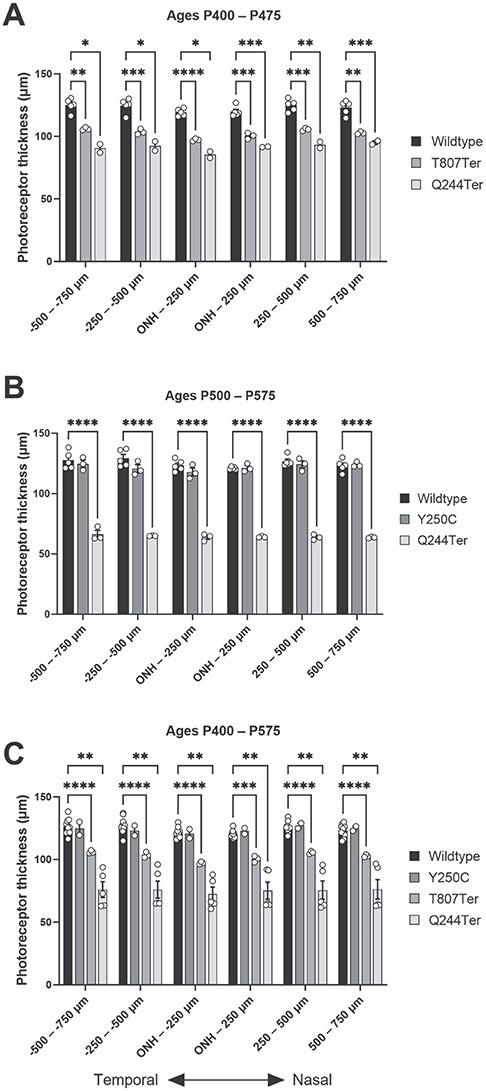


In the legend, sections of the second and third sentences should read: ``[...]mice are 17% and 26% lower than those of wildtype (n = 5) mice, respectively. (B) Measurements of photoreceptor layer thickness in P500–575 Impg2Q244Ter/Q244Ter (n = 3) mice are 49% lower than those of wildtype mice, [...]'' instead of: ``[...]mice are ~16% and ~26% lower than those of wildtype (n = 5) mice, respectively. (B) Measurements of photoreceptor layer thickness in P500–575 Impg2Q244Ter/Q244Ter (n = 3) mice are ~48% lower than those of wildtype mice, [...]''.

These errors have been emended in the article

