# Supplementary material for: Heterogeneity in the progression of retinal pathologies in mice harboring patient mimicking Impg2 mutations
Source: Hum Mol Genet. 2023 Nov 17;33(5):448–64. doi: 10.1093/hmg/ddad199 (PMC10877459; doi:10.1093/hmg/ddad199)
Supplement: impg2_supplement_2_clean_ddad199 [file impg2_supplement_2_clean_ddad199.pdf]

SUPPLEMENTARY FIGURES

Figure S1

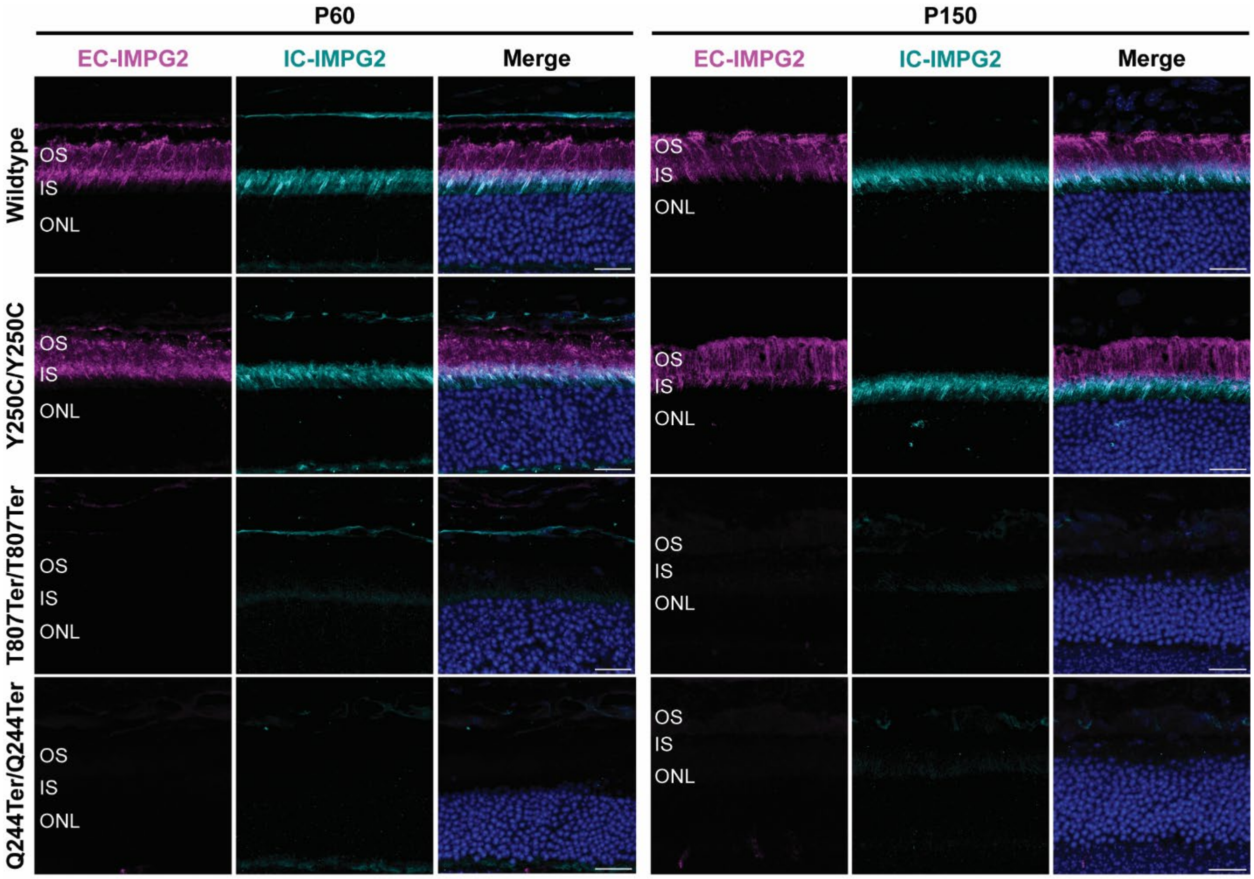

Supplementary Figure 1. Changes in IMPG2 expression at P60 and 150 in *Impg2* model mice. Confocal micrographs showing immunofluorescent labeling of the IMPG2 extracellular epitope (EC-IMPG2; magenta), intracellular epitope (IC-IMPG2; cyan), and DAPI (labels nuclei; blue) in the peripheral retina of P60 and P150 WT, *Impg2*$^{Y250C/Y250C}$, *Impg2*$^{T807Ter/T807Ter}$, and *Impg2*$^{Q244Ter/Q244Ter}$ mice. During aging, WT and *Impg2*$^{Y250C/Y250C}$ mice exhibit EC-IMPG2 staining across the OS and in the outer part of the IS. IC-IMPG2 labeling is observed in the IS. IMPG2 immunofluorescence was absent in *Impg2*$^{T807Ter/T807Ter}$ and *Impg2*$^{Q244Ter/Q244Ter}$ mice.

Figure S2

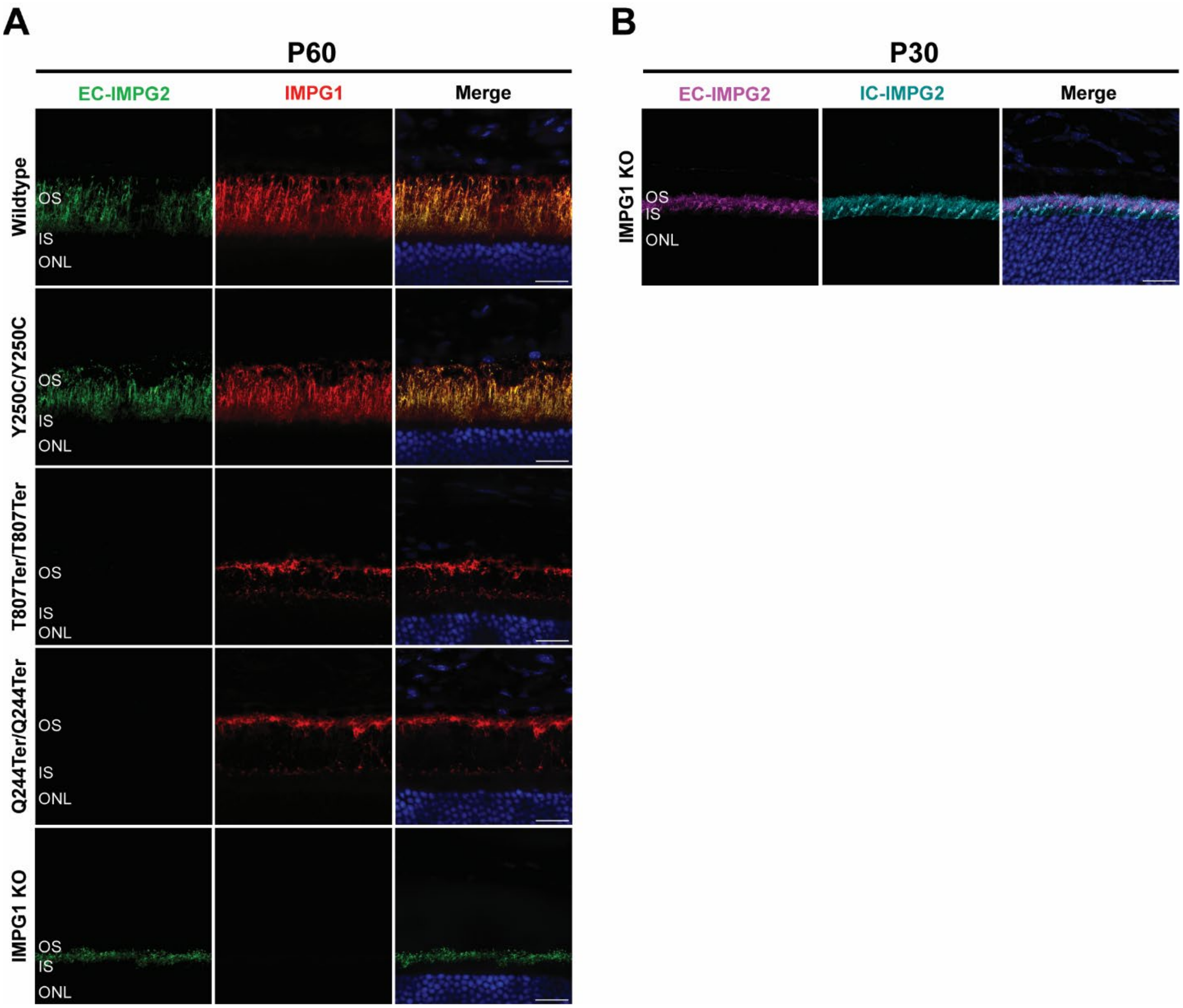

Supplementary Figure 2. Impact of IMPG2 mutant mice on IMPG1 expression. (A) Confocal micrographs showing immunofluorescent labeling of the IMPG2 extracellular epitope (EC-IMPG2; green), IMPG1 (red), and DAPI (blue) in the peripheral retina of P60 WT, *Impg2*$^{Y250C/Y250C}$, *Impg2*$^{T807Ter/T807Ter}$, *Impg2*$^{Q244Ter/Q244Ter}$, and *Impg1* KO mice. IMPG1 expression was reduced and mislocalized in *Impg2*$^{T807Ter/T807Ter}$ and *Impg2*$^{Q244Ter/Q244Ter}$ mice and absent in *Impg1* KO mice. **(B)** Immunofluorescent labeling of the IMPG2 extracellular epitope (EC-IMPG2; magenta), intracellular epitope (IC-IMPG2; cyan), and DAPI (blue) in the peripheral retina of P60 *Impg1* KO mice showing loss of the EC-IMPG2 at the OS level.

Figure S3

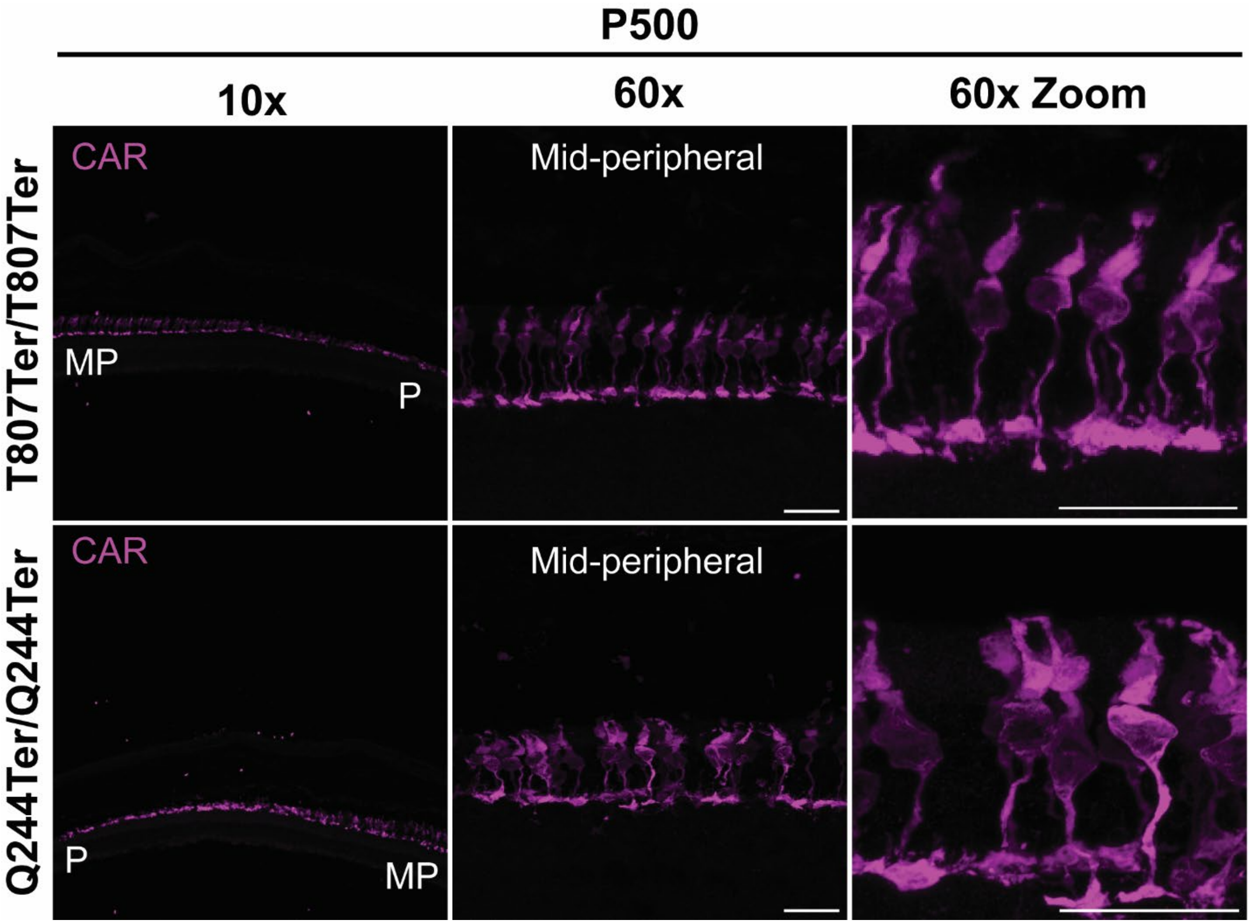

Supplementary Figure 3. Differences in cone photoreceptor morphology in mid-peripheral and peripheral retinas of P500 *Impg2$^{Q244Ter/Q244Ter}$* and *Impg2$^{T807Ter/T807Ter}$* mice. Immunofluorescent labeling of cone arrestin (CAR, magenta) in the mid-peripheral retina of P500 *Impg2$^{T807Ter/T807Ter}$* and *Impg2$^{Q244Ter/Q244Ter}$* mice. Cone photoreceptors were better preserved in the mid-peripheral retina than in the peripheral area. Images were acquired at different magnifications (10x and 60x) to illustrate differences in peripheral (P) and mid-peripheral (MP) CAR staining. Scale bar, 20 μm.

Figure S4

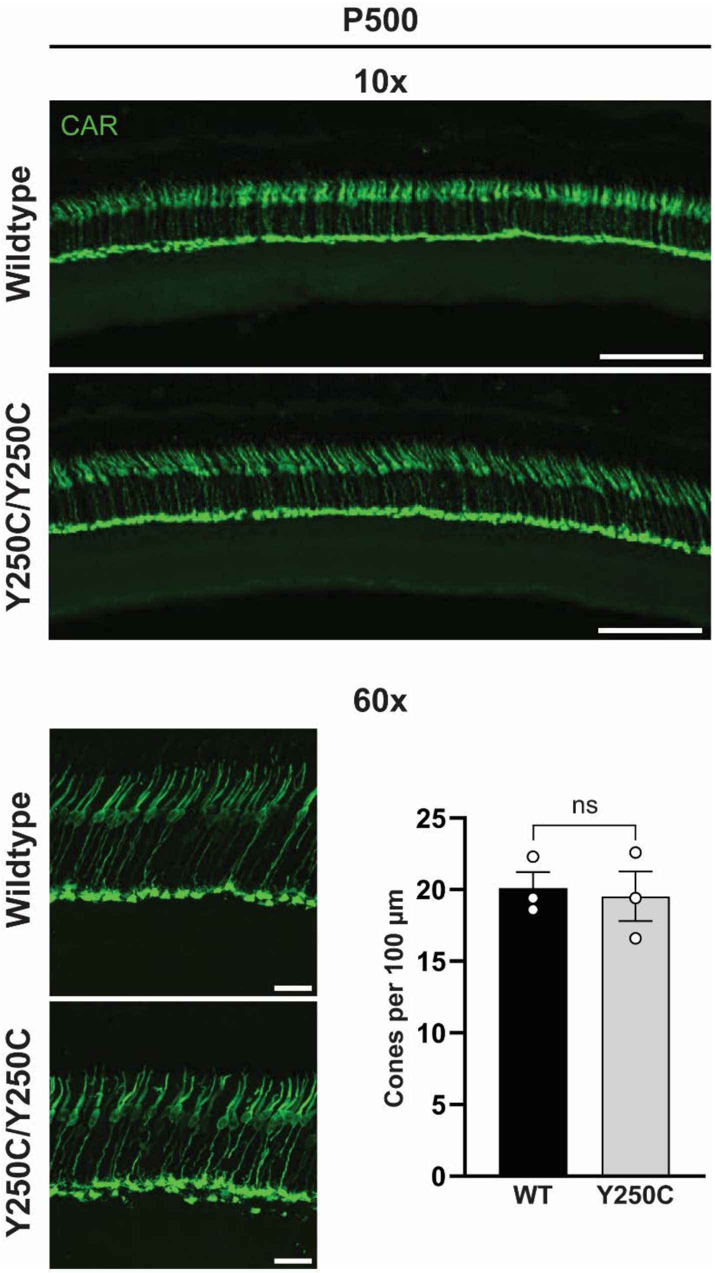

Supplementary Figure 4. Wildtype and *Impg2*$^{Y250C/Y50C}$ retinas contain equivalent numbers of cones*.* Immunofluorescent labeling of cone arrestin (CAR) in wildtype (WT) and *Impg2*$^{Y250C/Y250C}$ retinal sections at 10x and 60x magnifications. Quantification of cones in 60x images (n = 3 mice/genotype), acquired in the peripheral retina, demonstrated that this region contains 20.1 ± 1.1 cones in WT mice and 19.5 ± 1.7 cones in *Impg2*$^{Y250C/Y250C}$ mice per 100 µm (P = 0.80). Representative 60x images are from different sections than those shown from the 10x images. Scale bars, 100 µm (10x) and 20 µm (60x). Error bars represent the SEM. Statistical significance was determined by an unpaired t-test. ns = non-significant.

Figure S5

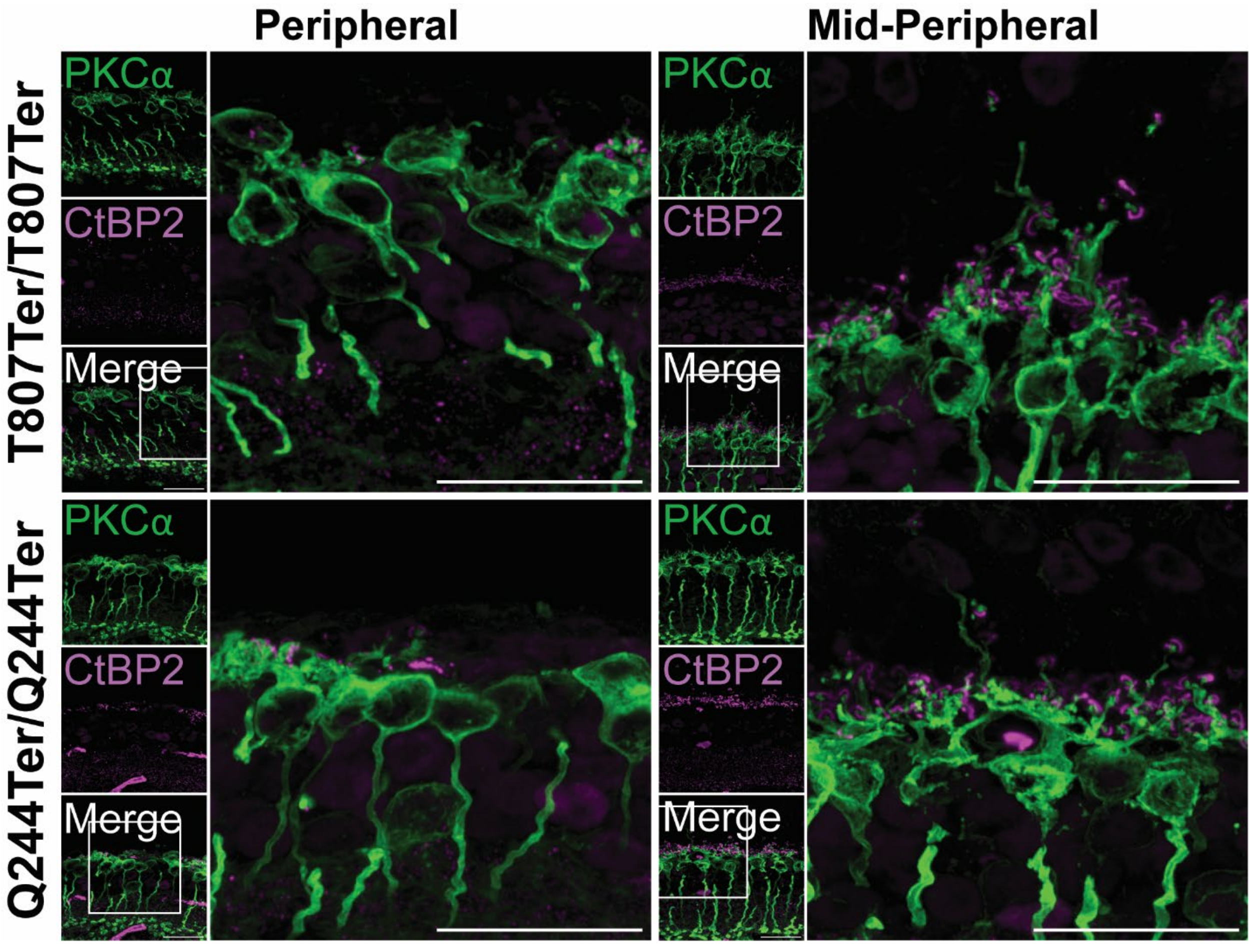

Supplementary Figure 5. Differences in ribbon synapses between mid-peripheral retina and peripheral retina in *Impg2*$^{T807Ter/T807Ter}$ and *Impg2*$^{Q244Ter/Q244Ter}$ mice at P500. Immunofluorescent labeling of PKCα (green, labels rod bipolar cells) and CtBP2 (magenta, labels ribbon synapses) at P500 in the retina of *Impg2*$^{T807Ter/T807Ter}$ and *Impg2*$^{Q244Ter/Q244Ter}$ mice. Ribbon synapses in the mid-peripheral retina of *Impg2*$^{T807Ter/T807Ter}$ and *Impg2*$^{Q244Ter/Q244Ter}$ mice at P500 showed preserved synaptic contacts compared to the peripheral outer plexiform layer. Images were acquired from mid-peripheral retinas. Inset locations are approximate. Scale bar, 20 μm.
